# Targeted Delivery of mRNA to the Heart via Extracellular Vesicles or Lipid Nanoparticles

**DOI:** 10.1002/jev2.70324

**Published:** 2026-06-11

**Authors:** Muhammad Nawaz, Benyapa Tangruksa, Sepideh Heydarkhan‐Hagvall, Franziska Kohl, Hernán González‐King Garibotti, Yujia Jing, Zahra Payandeh, Azadeh Reyahi, Karin Jennbacken, John Wiseman, Leif Hultin, Lennart Lindfors, Jane Synnergren, Hadi Valadi

**Affiliations:** ^1^ Department of Rheumatology and Inflammation Research Institute of Medicine Sahlgrenska Academy University of Gothenburg Gothenburg Sweden; ^2^ Chief Medical Office Global Patient Safety BioPharmaceuticals R&D AstraZeneca Gaithersburg Maryland USA; ^3^ Systems Biology Research Center School of Bioscience University of Skövde Skövde Sweden; ^4^ Centre for Genomics Research Discovery Sciences BioPharmaceuticals R&D AstraZeneca Gothenburg Sweden; ^5^ Department of Medical Biochemistry and Biophysics Karolinska Institute Solna Stockholm Sweden; ^6^ Bioscience Cardiovascular Research and Early Development Cardiovascular, Renal and Metabolism (CVRM) BioPharmaceuticals R&D AstraZeneca Gothenburg Sweden; ^7^ Advanced Drug Delivery Pharmaceutical Sciences BioPharmaceuticals R&D AstraZeneca Gothenburg Sweden; ^8^ Discovery Imaging Clinical Pharmacology and Safety Sciences BioPharmaceuticals R&D AstraZeneca Gothenburg Sweden; ^9^ Department of Molecular and Clinical Medicine Institute of Medicine Sahlgrenska Academy University of Gothenburg Gothenburg Sweden

**Keywords:** extracellular vesicles, lipid nanoparticles, systemic administration, targeted mRNA delivery, VEGF‐A

## Abstract

Efficient and specific delivery of mRNA to target tissues is critical for maximising therapeutic benefits while minimising off‐target effects and systemic toxicity. Systemic administration of mRNA using lipid nanoparticles (LNPs) or extracellular vesicles (EVs) typically leads to predominant accumulation in the liver. We hypothesised that cardiac‐specific EVs could promote enhanced relative cardiac enrichment of delivered mRNA compared with non‐cardiac EVs or LNPs. In mice, intravenous administration of cardiac progenitor cell‐derived EVs (CPC‐EVs) achieved the greatest relative cardiac selectivity of modified mRNA encoding vascular endothelial growth factor A (VEGF‐A) to the heart, with reduced liver accumulation relative to non‐cardiac EVs and LNPs. Cytokine profiling across seven organs revealed that LNP delivery triggered a widespread pro‐inflammatory response, whereas CPC‐EVs elicited only a localised and limited cytokine activation, suggesting a more favourable safety profile. Furthermore, direct intramyocardial injection of CPC‐EVs not only led to efficient mRNA uptake by cardiac tissue and robust VEGF‐A protein expression, but also minimal transcriptomic perturbation in the cardiac tissue, as confirmed by RNA‐seq. In contrast, LNPs and non‐cardiac EVs induced widespread perturbation in the transcriptome of cardiac tissue. Functionally, *VEGF‐A* mRNA delivery via CPC‐EVs markedly increased CD31 and α‐SMA expression and vessel formation in ex vivo aortic ring assays, confirming enhanced angiogenic potential. Together, these findings support CPC‐EVs as a promising platform for achieving enhanced cardiac delivery of mRNA, with reduced liver accumulation, limited off‐target transcriptomic perturbation, a more selective cytokine response, and enhanced angiogenic activity in ex vivo assays.

## 1 Introduction

Despite decades of progress in RNA‐based therapeutics, the development of delivery systems capable of targeting mRNA delivery to specific organs or cell types remains a major challenge. Targeted delivery is essential to maximise the therapeutic impact of drugs by ensuring their direct transport to the specific site of need, thereby enhancing efficacy and reducing the required dosage (Li et al. 2023). This focused drug delivery approach not only promises improved treatment outcomes but also diminishes the likelihood of unintended side effects and systemic toxicity (Tewabe et al. 2021). Among vital organs, the heart presents unique delivery barriers due to its dynamic environment, limited permeability and high perfusion, making it particularly difficult to achieve effective mRNA localisation and translation within the targeted cardiac tissue.

Lipid nanoparticles (LNPs) and extracellular vesicles (EVs) have emerged as leading non‐viral platforms for RNA delivery. However, both tend to accumulate in the liver following systemic administration (Wiklander et al. 2015; Rohner et al. 2022; Paunovska et al. 2019; Kim et al. 2021; Gupta et al. 2021). This severely limits their therapeutic utility for extrahepatic targets such as the heart. Overcoming this hepatic tropism necessitates the development of organ‐ or cell‐specific delivery strategies capable of directing RNA cargo selectively to cardiac tissue, as EV biodistribution is strongly influenced by their cellular origin and delivery route (Wiklander et al. 2015).

The delivery of mRNA encoding vascular endothelial growth factor A (VEGF‐A) to cardiac tissue has emerged as a promising strategy for treating cardiovascular diseases. By promoting neovascularisation (angiogenesis), this approach offers potential therapeutic benefits, particularly for patients with heart failure (Anttila et al. 2020; Collen et al. 2022). The therapeutic application of VEGF‐A protein to stimulate vascularisation has been investigated for decades in both preclinical models and clinical trials (Gaffney et al. 2007; Giacca and Zacchigna 2012; Yla‐Herttuala 2013).

The pioneering work has shown that chemically modified *VEGF‐A* mRNA can achieve strong, yet transient protein expression lasting several days in mice, large animal models and human trials (Collen et al. 2022; Zangi et al. 2013; Carlsson et al. 2018; Gan et al. 2019; Sun et al. 2018; Pehrsson et al. 2019; Chien et al. 2014). However, these first‐in‐human clinical studies relied on direct intramyocardial injections of unencapsulated *VEGF‐A* mRNA, without the use of delivery vehicles, such as LNPs. This limits the potential for systemic or minimally invasive administration and highlights the need for effective, heart‐targeted RNA delivery systems. Although localised, minimally invasive administration of naked mRNA, such as direct intramyocardial injection, has been explored in early studies, systemic delivery requires the use of RNA packaging systems to protect the cargo during delivery (Rohner et al. 2022). Current RNA carriers such as LNPs have been primarily optimised for delivery efficiency, with limited success in achieving cell‐ or tissue‐specific targeting. Multiple studies have reported that systemically administered mRNA, whether delivered via LNPs or EVs, tends to accumulate predominantly in the liver due to hepatic clearance through the circulatory system (Wiklander et al. 2015; Gupta et al. 2021).

EVs are a heterogeneous population of lipid bilayer‐enclosed vesicles, including exosomes and microvesicles, that are secreted by nearly all cell types. EVs are present in body fluids and conditioned media from cultured cells (Nawaz et al. 2016). EVs play a key role in intercellular communication (Simons and Raposo 2009) by transporting biomolecules such as RNA, lipids and proteins between cells and organs (Vella et al. 2007).

In this study, we performed a head‐to‐head comparison between cardiac progenitor cell‐derived EVs (CPC‐EVs, cardiac‐specific EVs), non‐cardiac EVs and LNPs to evaluate both the relative biodistribution and delivery efficiency of mRNA to the heart. Our results demonstrated that CPC‐EVs enable enhanced and relatively selective cardiac delivery of therapeutic mRNA, with reduced hepatic accumulation. Moreover, CPC‐EVs facilitated robust expression of the encoded protein, VEGF‐A, in cardiac tissue, while inducing the fewest off‐target transcriptomic alterations relative to other tested delivery platforms. CPC‐EVs elicited a tissue‐selective and limited systemic cytokine response relative to the broad pro‐inflammatory activation triggered by LNPs, indicating a potentially improved safety profile of CPC‐EVs. These findings position CPC‐EVs as a superior vehicle for heart‐targeted mRNA therapy.

## 2 Results

### 2.1 Delivery of *VEGF‐A* mRNA to Cells via Lipid Nanoparticles

The composition and chemical structure of DLin‐MC3‐DMA LNPs used for mRNA delivery to cells in this study are shown in Figure S1A. The sequence of the sense strand of the chemically modified *VEGF‐A* mRNA, along with the corresponding protein sequence, is provided in Figure S1B,C, respectively.

The cellular uptake and translation of *VEGF‐A* mRNA delivered via LNPs were assessed in vitro (Figure S2A). Three human cell types were examined: cardiac progenitor cells (CPCs), human umbilical vein endothelial cells (HUVECs) and lung epithelial cells (HTBs). 24 h after LNP internalisation, *VEGF‐A* mRNA levels were quantified in the lysates of all three recipient cell types, and secreted VEGF‐A protein levels were measured in the supernatants of corresponding cells. The results showed that all three cell types internalised significant amounts of LNP‐delivered *VEGF‐A* mRNA and translated it into substantial levels of VEGF‐A protein (Figure S2B,C).

### 2.2 Cytoplasmic Delivery of Translatable mRNAs via Extracellular Vesicles

#### 2.2.1 Incorporation of Cy5‐eGFP mRNA and VEGF‐A mRNA into Extracellular Vesicles

**FIGURE 1:**
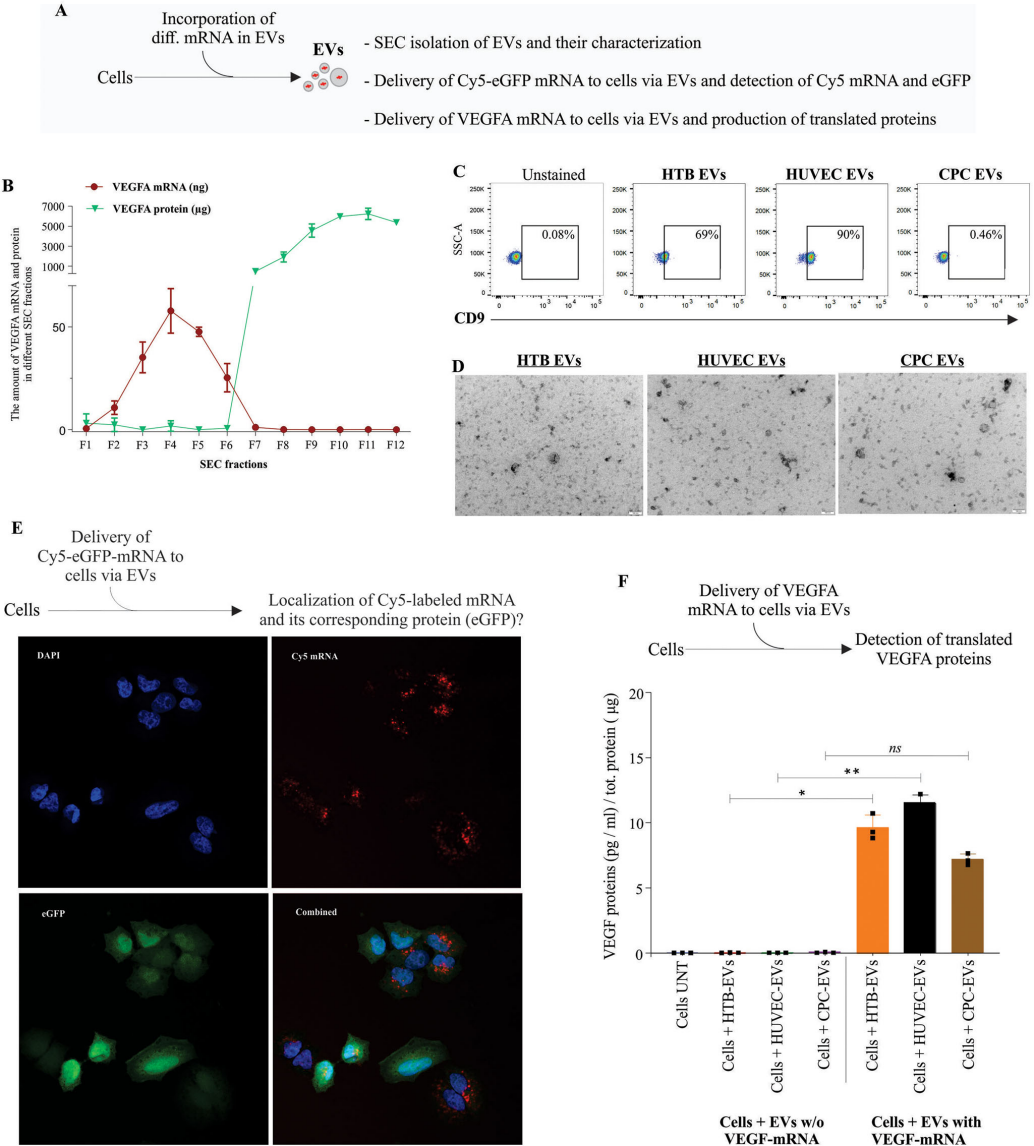
Cytoplasmic delivery of translatable mRNAs via extracellular vesicles. (A) Schematic overview of extracellular vesicle (EV)‐mediated delivery and analysis of translatable mRNAs. Following 24 h of LNP‐mRNA uptake by cardiac progenitor cells (CPCs), human umbilical vein endothelial cells (HUVECs), and a human lung epithelial cell line (HTB), conditioned media were collected, and EVs were isolated by size exclusion chromatography (SEC). Twelve collected SEC fractions were analysed for the presence of *VEGF‐A* mRNA and VEGF‐A protein. (B) *VEGF‐A* mRNA was predominantly detected in early SEC fractions (F1–F6), whereas free VEGF‐A protein was enriched in later fractions (F7–F12), as determined by qPCR and ELISA, respectively. (C) Flow cytometric characterisation of EV markers CD63 and CD9 in pooled SEC fractions (F1–F6). EVs were first captured using an anti‐CD63 antibody, followed by detection with a PE‐conjugated anti‐CD9 antibody. Unstained controls showed a negligible signal. (D) Representative transmission electron microscopy images of HTB‐EVs, HUVEC‐EVs and CPC‐EVs. Scale bar: 100 nm. (E) Visualisation of EV‐mediated delivery of fluorescently labelled translatable mRNA in vitro. SEC‐EVs containing Cy5‐eGFP mRNA were added to recipient HTB cells. After 24 h of incubation, cytoplasmic localisation of Cy5‐labelled mRNA (red) and its translation into eGFP (green) were visualised by confocal microscopy. Representative images are shown. (F) Quantification of VEGF‐A protein production following EV‐mediated delivery of VEGF‐A mRNA in vitro (pg/mL)/total protein (µg). *VEGF‐A* mRNA‐loaded EVs derived from cardiac progenitor cells (CPC‐EVs), human umbilical vein endothelial cells (HUVEC‐EVs), and HTB cells (HTB‐EVs) were used. An equal amount of *VEGF‐A* mRNA (550 ng) was delivered to recipient HUVECs via each EV type. EVs without *VEGF‐A* mRNA loading, as well as untreated recipient cells (UNT), were used as controls. At 24 h post‐treatment, supernatants were collected and analysed for VEGF‐A protein using ELISA. Elevated VEGF‐A protein levels were detected in cells treated with *VEGF‐A* mRNA‐loaded EVs, whereas minimal or undetectable levels were observed in untreated cells and in cells treated with EVs lacking mRNA cargo. VEGF‐A protein levels are expressed as pg/mL and normalised to total protein content where indicated. Statistical comparisons among groups were performed using the Kruskal–Wallis test followed by Dunn's multiple‐comparison test to compare mRNA‐loaded EVs with their corresponding unloaded EV controls. Statistical significance is indicated as *p < 0.05 and **p < 0.01; ns, not significant. Data are presented as mean ± SD of *n* = 3 biological replicates per group. UNT, untreated; w/o, without.

Previously, we have shown that the LNP‐delivered mRNA is incorporated into EVs after endosomal escape within recipient cells (Nawaz et al. 2023; Maugeri et al. 2019). Leveraging this natural endocytosis‐exocytosis route of LNP‐mRNA secretion, Cy5‐labelled *eGFP* mRNA and *VEGF‐A* mRNA were incorporated into EVs (Figure 1A; Figure S2A). Cy5‐labelled mRNA was loaded in EVs from HTB cells, whereas the *VEGF‐A* mRNA was loaded into three types of EVs secreted from CPCs (CPC‐EVs), HUVECs (HUVEC‐EVs) and HTBs (HTB‐EVs).

#### 2.2.2 Purification of mRNA‐Loaded EVs and Quantitative Analysis of mRNA in SEC‐EV Fractions

To isolate mRNA‐containing EVs, culture supernatants were collected after treatment, and EVs were purified using size exclusion chromatography (SEC). First, in the HTB‐derived EVs, after the void volume had passed, 12 SEC fractions were collected and analysed for the presence of *VEGF‐A* mRNA (via qPCR) and VEGF‐A protein (via ELISA) (Figure S2A). Our data show that the *VEGF‐A* mRNA was detected predominantly in the early six fractions (F1–6), whereas VEGF‐A protein was confined to later fractions (F7–12), suggesting compartmentalisation of mRNA cargo (Figure 1B). To assess mRNA secretion dynamics, a time‐course analysis was performed following LNP‐mediated delivery of *VEGF‐A* mRNA. A continuous release of *VEGF‐A* mRNA into EVs was observed, peaking at 5 h post‐treatment, with the highest levels detected in fraction 4 (Figure S3A,B).

*VEGF‐A* mRNA was also examined in EV SEC fractions (F1–6) derived from CPCs and HUVECs. Significant levels of *VEGF‐A* mRNA were detected in CPC‐EVs and HUVEC‐EVs after 24 h of treatment, compared to negligible levels in EVs from corresponding untreated cells (unloaded EVs) (Figure S3C–E). In an additional experiment, the first six SEC fractions (F1–6) were pooled, total RNA was isolated, and *VEGF‐A* mRNA quantification (via qPCR), confirming robust enrichment of *VEGF‐A* mRNA across all three EV types (Figure S3F).

#### 2.2.3 Characterisation of Extracellular Vesicles

Based on the manufacturer's specifications for qEV70/10 mL columns (Izon Science), the first four 5 mL SEC fractions typically contain EVs when 10 mL of media is loaded. In our setup, since 15 mL of conditioned media was loaded, two extra fractions (i.e., six 5 mL SEC fractions) were collected for EV characterisation. To confirm EV identity, pooled samples of F1–6 were subjected to immunoaffinity capture using anti‐CD63 and anti‐CD9 antibodies, followed by flow cytometry analysis. EVs derived from HTBs and HUVECs, which were positive for CD63, were also positive for CD9, showing dual positivity for EV markers, whereas CPC‐EVs were positive for CD63 but negative for CD9 (Figure 1C). Specifically, among CD63^+^ EVs, 69% of HTB‐EVs and 90% of HUVEC‐EVs were also CD9^+^, while only 0.46% of CPC‐EVs co‐expressed CD9.

The morphology of EVs obtained from three different cell types was examined by transmission electron microscopy (Figure 1D). The nanoparticle tracking analysis (NTA) further determined the size distribution and concentration of the EVs, revealing mean diameters ranging from 149 to 158 nm, and mode sizes between 146 and 152 nm (Figure S4).

#### 2.2.4 mRNA‐Loaded EVs Purified by Size Exclusion Chromatography Retain Their Ability to Deliver Intact Exogenous mRNAs to Cells

To confirm that EV‐mediated delivery preserves the integrity and translatability of mRNA cargo, we employed a fluorescent reporter system. HTB SEC‐EVs loaded with Cy5‐labelled *eGFP* mRNA were delivered to HTB‐177 cells. 24 h after delivery, both Cy5‐labelled mRNA and the translated eGFP protein were primarily localised in the cytosol, confirming successful cytoplasmic delivery and translation of intact mRNA (Figure 1E).

The mRNA delivery efficiency of SEC‐EVs was further evaluated for functional mRNA. Three types of SEC‐EVs (CPC‐EVs, HUVEC‐EVs and HTB‐EVs) containing *VEGF‐A* mRNA were applied to recipient endothelial cells (HUVECs), and translation efficiency was assessed 24 h post‐treatment. All three SEC‐EV types successfully delivered functional *VEGF‐A* mRNA, as evidenced by significant VEGF‐A protein production in treated cells compared to untreated controls or cells treated with unloaded EVs (Figure 1F).

Together, these findings demonstrate that SEC‐EVs efficiently transport exogenous mRNA into recipient cells, where it remains intact and functional, leading to de novo protein synthesis.

### 2.3 Intravenous and Intramuscular Delivery of Luciferase mRNA via SEC‐EVs or LNPs

**FIGURE 2:**
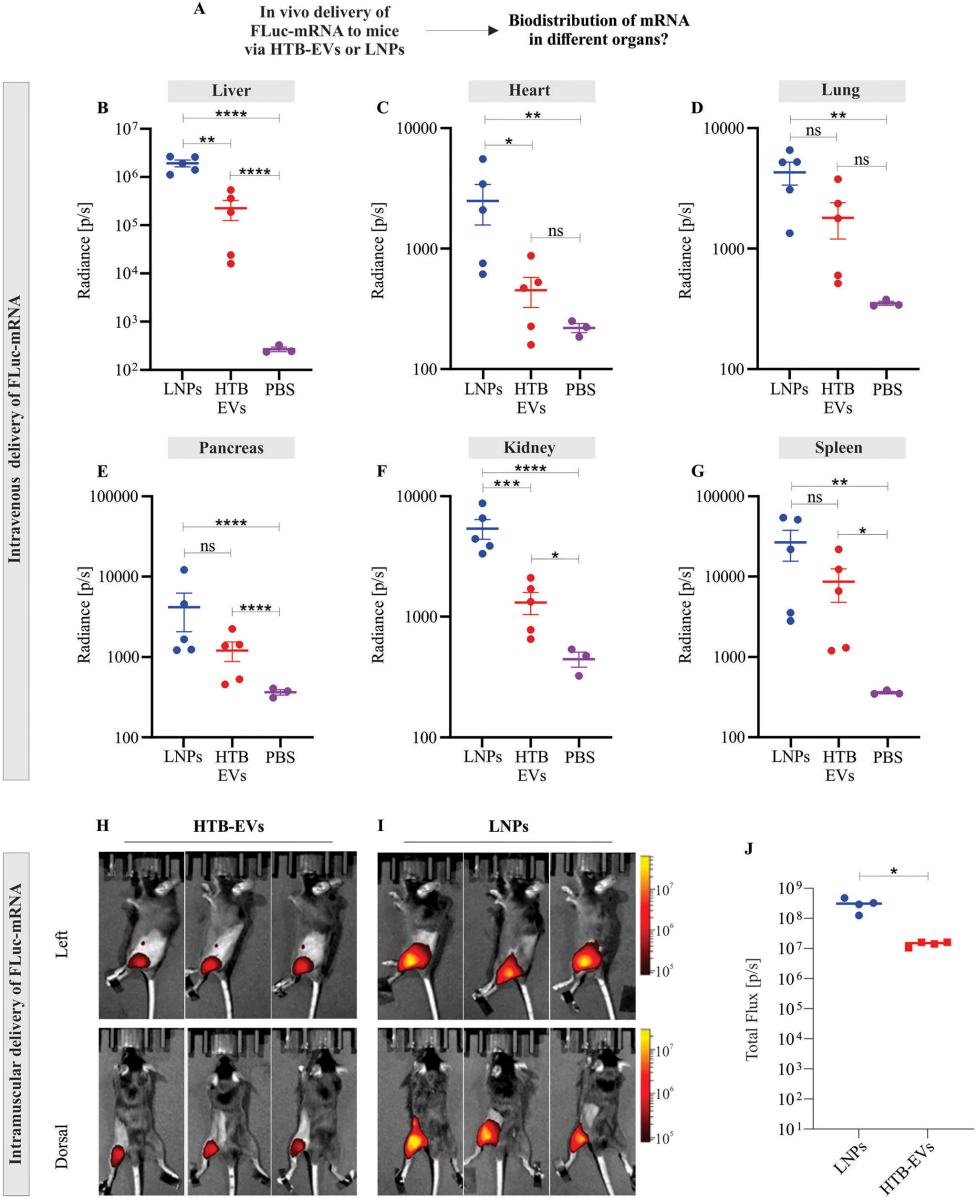
Intramuscular and intravenous delivery of luciferase mRNA via extracellular vesicles (EVs) or lipid nanoparticles (LNPs). (A) Schematic representing in vivo administration of firefly luciferase mRNA (FLuc‐mRNA) via HTB‐derived extracellular vesicles (HTB‐EVs) and assessment of biodistribution. Mice were intravenously injected with HTB‐EVs or LNPs containing FLuc‐mRNA (1 µg in 100 µL). To evaluate translation of FLuc‐mRNA into luciferase protein, a D‐luciferin (5 mL/kg) was administered intravenously 6 h post‐injection. Mice were euthanised 20 min after luciferin administration, and organs were dissected and imaged using an IVIS Spectrum within 5 min of euthanasia. Total radiance [p/s] (luciferase‐luciferin activity) was quantified as a measure of luciferase activity derived from delivered FLuc‐mRNA. Quantification of luciferase radiance in (B) liver, (C) heart, (D) lung, (E) pancreas, (F) kidney and (G) spleen. Statistical comparisons among groups were performed using one‐way ANOVA with appropriate multiple‐comparisons correction. Statistical significance is indicated as *p < 0.05, **p < 0.01, ***p < 0.001 and ****p < 0.0001; ns, not significant, for the indicated comparisons. Data are presented as mean ± SEM of *n* = 5 biological replicates per treatment group, except for untreated (PBS) controls where *n* = 3. In a separate experiment, EVs or LNPs containing FLuc‐mRNA (215 ng, in 30 µL) were administered intramuscularly to female C57bl/Ncr mice. To evaluate translation of FLuc‐mRNA into luciferase protein in the muscles, a D‐luciferin (5 mL/kg) was administered intravenously 6 h post‐injection. Injection sites were imaged using an IVIS Spectrum within 5 min of luciferin administration. Total radiance (luciferase‐luciferin activity) was quantified in dorsal and left muscle after FLuc‐mRNA delivery via (H) HTB‐EVs, and (I) LNPs. (J) Total flux (radiance) of luciferase quantified after FLuc‐mRNA delivery via HTB‐EVs or LNPs. Statistical comparisons were performed using a Mann‐Whitney U test to evaluate differences in luciferase total flux between HTB‐EVs and LNP groups. Statistical significance is indicated as *p < 0.05. Data are presented as mean ± SEM of *n* = 4 biological replicates per treatment group.

To assess whether SEC‐isolated EVs retain the capacity to deliver translatable exogenous mRNA in vivo, we first performed a proof‐of‐concept experiment using a reporter transcript. Firefly luciferase mRNA (Fluc‐mRNA) was administered intravenously to mice using HTB‐derived SEC‐EVs, with LNPs included as a reference formulation (Figure 2A). This experiment was designed to establish in vivo translation of EV‐delivered mRNA rather than to evaluate tissue‐specific targeting. Following intravenous delivery via EVs or LNPs, the biodistribution and translation of delivered mRNA were evaluated across different tissues. Although LNPs exhibited higher overall delivery efficiency and luciferase expression compared to HTB‐EVs, neither vehicle demonstrated strong tissue‐specific targeting, with luciferase activity broadly distributed across tissues (Figure 2B–G, organ‐wise quantification). To evaluate uptake by difficult‐to‐transfect cell types such as muscle cells, intramuscular (IM) injections of Fluc‐mRNA were performed using EVs or LNPs. In vivo imaging system (IVIS) analysis confirmed effective uptake and translation in muscle cells, with detectable luciferase expression at the injection site (Figure 2H–J, IM delivery and quantification).

### 2.4 Cardiac Progenitor Cell–Derived EVs Exhibit Targeted Delivery of mRNA to the Heart

Systemic administration of molecular therapies, including RNA‐based therapeutics, frequently results in hepatic accumulation due to first‐pass clearance by the liver via the circulatory system, a phenomenon previously shown to depend on EV cell source and administration route (Wiklander et al. 2015). Consequently, the development of delivery vehicles capable of reducing hepatic accumulation and enhancing extrahepatic delivery of mRNA remains a critical challenge. Our findings suggest that cardiac‐specific EVs (CPC‐EVs) exhibit reduced hepatic accumulation and enhanced relative enrichment of exogenous *VEGF‐A* mRNA in the heart compared to LNPs and non‐cardiac EVs.

**FIGURE 3:**
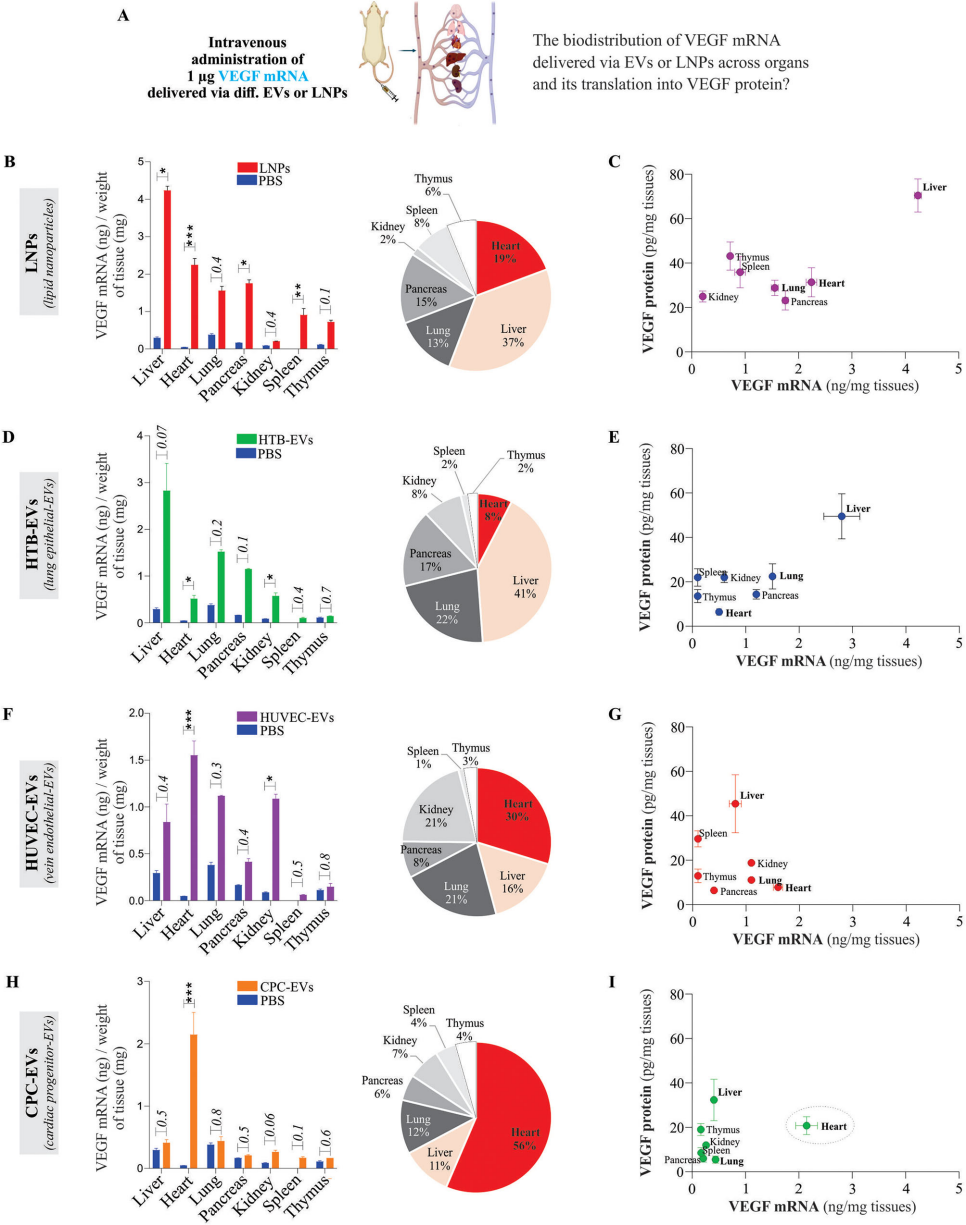
Relative organ distribution and tissue selectivity of EV‐ and LNP‐mediated *VEGF‐A* mRNA delivery following intravenous administration. (A) Schematic overview of intravenous administration of *VEGF‐A* mRNA to C57BL/6Ncrl mice via lipid nanoparticles (LNPs) or extracellular vesicles (EVs) derived from CPCs (CPC‐EVs), HTB cells (HTB‐EVs) or HUVECs (HUVEC‐EVs). At 6 h post‐injection, mice were euthanised, and *VEGF‐A* mRNA levels were quantified across major organs to assess biodistribution. Relative tissue distribution and selectivity were further evaluated by comparing *VEGF‐A* mRNA and corresponding protein levels across tissues. (B) Relative distribution (%) and absolute levels (ng/mg tissue) of VEGF‐A mRNA across organs following LNP‐mediated delivery. (C) Quantitative analysis of organ‐specific localisation demonstrating the relationship between *VEGF‐A* mRNA levels and corresponding VEGF‐A protein levels across organs following LNP‐mediated delivery, showing predominant liver accumulation. (D) Relative distribution (%) and absolute levels (ng/mg tissue) of *VEGF‐A* mRNA across organs following HTB‐EV‐mediated delivery. (E) Quantitative analysis of organ‐specific localisation demonstrating the relationship between *VEGF‐A* mRNA levels and corresponding VEGF‐A protein levels across organs following HTB‐EV‐mediated delivery. (F) Relative distribution (%) and absolute levels (ng/mg tissue) of VEGF‐A mRNA across organs following HUVEC‐EV‐mediated delivery. (G) Quantitative analysis of organ‐specific localisation demonstrating the relationship between *VEGF‐A mRNA* levels and corresponding VEGF‐A protein levels across organs following HUVEC‐EV‐mediated delivery. (H) Relative distribution (%) and absolute levels (ng/mg tissue) of *VEGF‐A* mRNA across organs following CPC‐EV‐mediated delivery. (**I)** Quantitative analysis of organ‐specific localisation demonstrating the relationship between *VEGF‐A* mRNA levels and corresponding VEGF‐A protein levels across organs following CPC‐EV‐mediated delivery, showing enhanced relative cardiac enrichment. Statistical comparisons among groups were performed using the Kruskal–Wallis test. Statistical significance is indicated as *p < 0.05, **p < 0.01 and ***p < 0.001; whereas exact *p* values are provided for non‐significant comparisons where indicated. Data are presented as mean ± SD of *n* = 3 biological replicates per group. *VEGF‐A* mRNA levels are expressed as ng/mg tissue, and VEGF‐A protein levels as pg/mg tissue.

To evaluate biodistribution and relative organ‐specific levels of *VEGF‐A* mRNA, the mRNA was intravenously (i.v.) administered to mice using four different delivery platforms: CPC‐EVs, HTB‐EVs, HUVEC‐EVs and LNPs. The mRNA levels were quantified across seven major organs: heart, liver, lung, kidney, pancreas, spleen and thymus (Figure 3A). When the relative distribution of *VEGF‐A* mRNA across organs was compared for each delivery vehicle, LNPs, HTB‐EVs and HUVEC‐EVs predominantly delivered mRNA to the liver and exhibited non‐specific and broad distribution across multiple organs, without clear tissue selectivity (Figure 3B,D,F). Unencapsulated (naked) mRNA also exhibited the highest accumulation in the liver, while still being distributed across multiple organs (Figure S5). In contrast, CPC‐EVs efficiently and preferentially delivered *VEGF‐A* mRNA to the heart, indicating a distinct tissue‐targeting capability (Figure 3H).

Based on the relative distribution of *VEGF‐A* mRNA across tissues, CPC‐EVs exhibited the highest proportion of *VEGF‐A* mRNA localised to the heart, with ∼56% of the total detected mRNA located in the heart, compared to ∼8% for HTB‐EVs, ∼19% for LNPs and ∼30% for HUVEC‐EVs. Given that minimising hepatic accumulation is critical for targeted mRNA delivery, CPC‐EVs exhibited the lowest relative liver distribution. Specifically, approximately 11% of the total VEGF‐A mRNA detected across tissues was distributed to the liver, compared to ∼41% for HTB‐EVs, ∼37% for LNPs and ∼16% for HUVEC‐EVs. These findings highlight the ability of CPC‐EVs to reduce liver tropism, a major barrier in targeted mRNA delivery, thereby enabling preferential cardiac distribution of therapeutic mRNA relative to other organs. This capability may be important for the development of targeted mRNA therapies, as reduced hepatic accumulation could enable more efficient and tissue‐selective extrahepatic delivery to target organs.

We next assessed the primary localisation of the translated VEGF‐A protein across mouse tissues following intravenous administration of mRNA. mRNA delivery via LNPs and non‐cardiac EVs resulted in predominant localisation of both *VEGF‐A* mRNA and protein in the liver (Figure 3C,E,G; Figure S5). In contrast, CPC‐EV‐mediated delivery led to predominant localisation of both *VEGF‐A* mRNA and protein in the heart (Figure 3I), further suggesting improved cardiac enrichment of mRNA delivery compared to the tested platforms.

**FIGURE 4:**
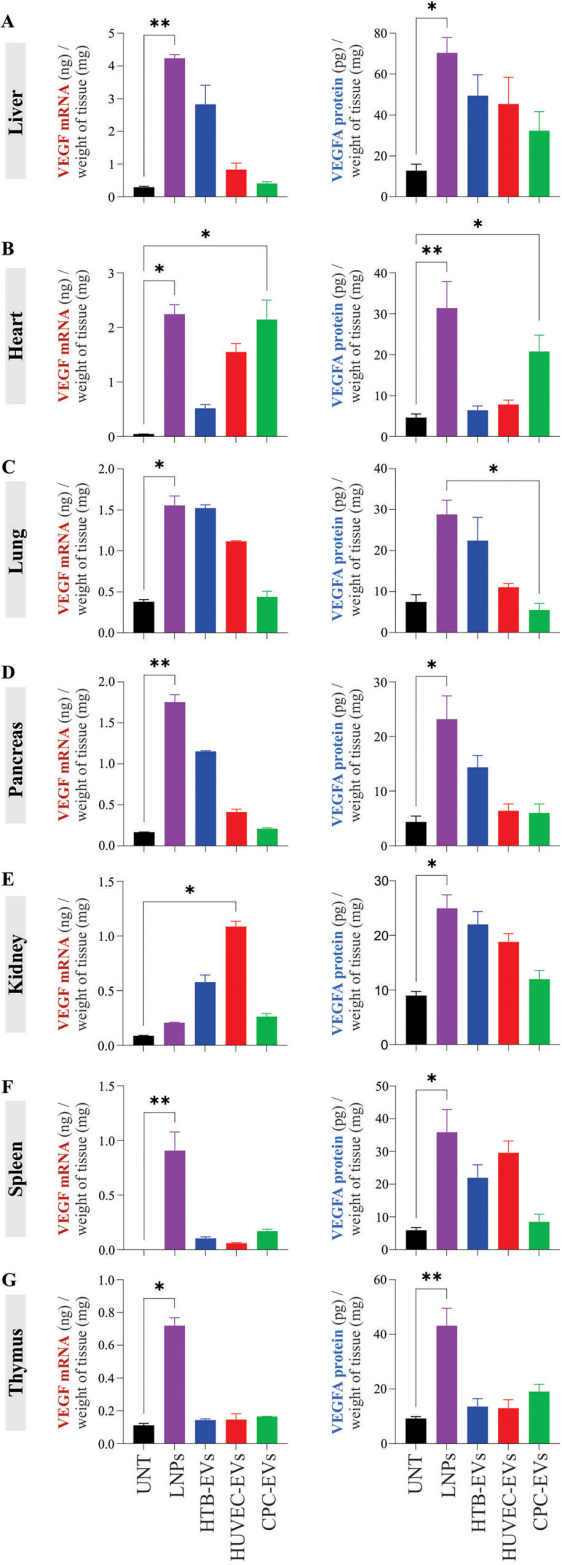
Head‐to‐head quantitative comparison of LNPs and EVs for *VEGF‐A* mRNA delivery and protein expression in vivo. C57BL/6Ncrl mice received an intravenous injection of 1 µg *VEGF‐A* mRNA formulated in CPC‐EVs, HTB‐EVs, HUVEC‐EVs or LNPs. Six hours after administration, mice were euthanised and organs were harvested for quantitative analysis of *VEGF‐A* mRNA and VEGF‐A protein levels using qPCR and ELISA, respectively. The figure presents absolute *VEGF‐A* mRNA and protein levels within individual organs following administration of equivalent mRNA doses across the different delivery platforms. Statistical comparisons among groups were performed using the Kruskal–Wallis test. Statistical significance is indicated as *p < 0.05 and **p < 0.01. Only statistically significant differences (*p* ≤ 0.05) are displayed. Data are presented as mean ± SD of *n* = 3 biological replicates per group. *VEGF‐A* mRNA levels are expressed as ng/mg tissue, and VEGF‐A protein levels as pg/mg tissue. UNT, untreated; LNPs, lipid nanoparticles; HTB‐EVs, HTB‐177 lung epithelial cell‐derived EVs; HUVEC‐EVs, human umbilical vein endothelial cell‐derived EVs; CPC‐EVs, cardiac progenitor cell‐derived EVs.

To directly compare the quantitative delivery efficiency of the different platforms, *VEGF‐A* mRNA and translated VEGF‐A protein levels were evaluated in each organ following delivery via CPC‐EVs, HTB‐EVs, HUVEC‐EVs or LNPs (Figure 4). Unlike Figure 3, which shows the relative distribution of mRNA across organs for each delivery platform, Figure 4 provides a head‐to‐head comparison of *VEGF‐A* mRNA and protein levels between delivery vehicles within the same organ. Overall, LNPs achieved the highest *VEGF‐A* mRNA and protein levels in most organs, particularly in the liver, lung, pancreas, spleen and thymus. In contrast, CPC‐EVs produced higher *VEGF‐A* mRNA and protein levels in the heart than HTB‐EVs and HUVEC‐EVs, indicating enhanced cardiac delivery efficiency among the EV‐based delivery platforms.

To further evaluate cardiac specificity, we calculated heart‐to‐organ ratios of *VEGF‐A* mRNA and protein levels following delivery via LNPs and the three EV types. These analyses demonstrated that CPC‐EVs achieved the highest heart‐to‐organ ratios across all evaluated tissues, indicating enhanced relative cardiac selectivity compared to LNPs and non‐cardiac EVs, which exhibited lower ratios consistent with broader and more liver‐predominant distribution profiles (Figure S6).

Together, these findings demonstrate that although LNPs achieved higher overall *VEGF‐A* mRNA and protein levels across multiple tissues, they lacked tissue selectivity. In contrast, CPC‐EVs exhibited pronounced relative cardiac enrichment, higher heart‐to‐organ ratios, and a more selective cardiac delivery profile with reduced off‐target distribution and lower hepatic accumulation compared to LNPs and non‐cardiac EVs.

### 2.5 Organ‐Specific Cytokine Responses Following Treatment With LNPs or CPC‐EVs

**FIGURE 5:**
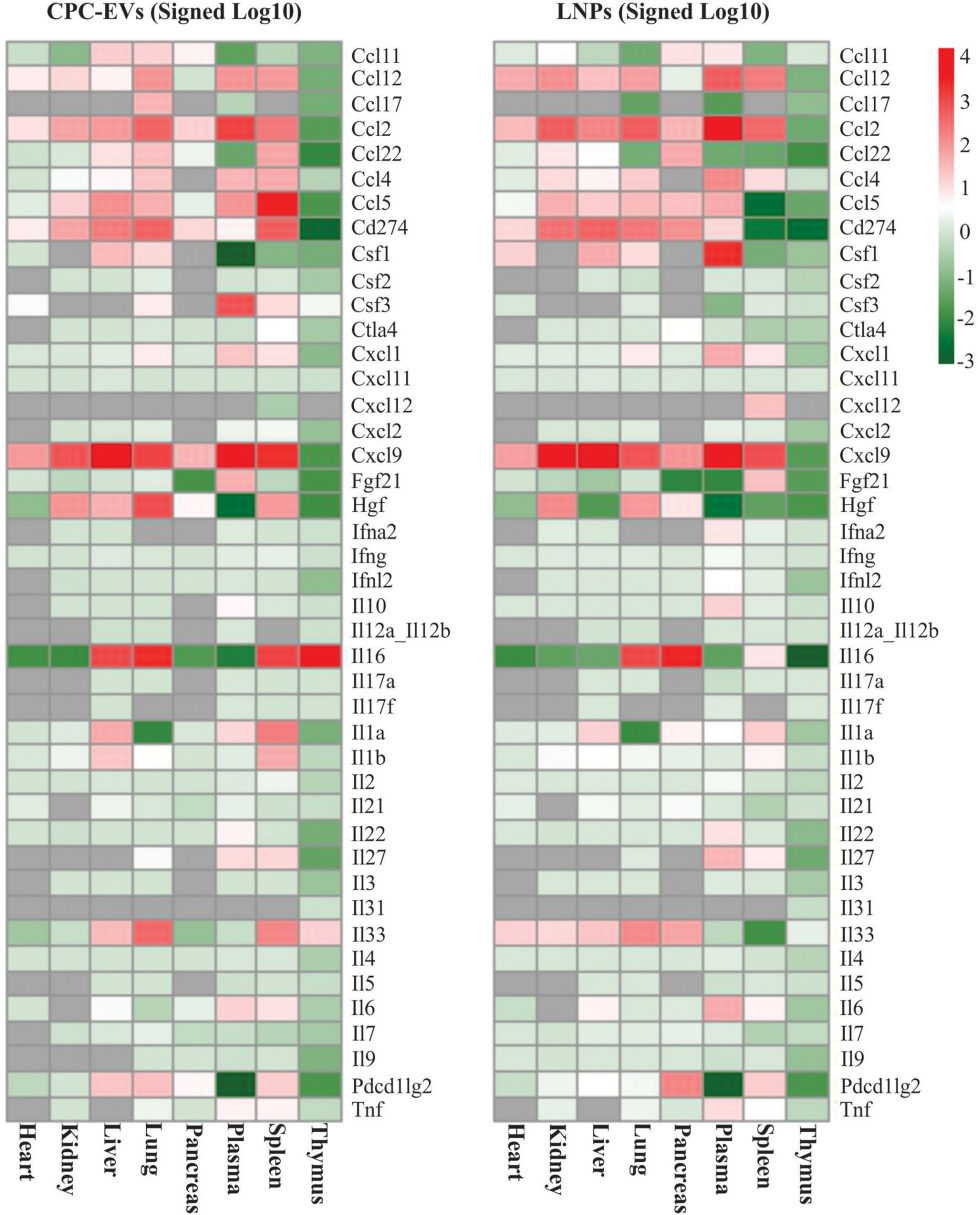
Organ‐specific cytokine responses following treatment with LNPs or CPC‐EVs. Heatmaps illustrating relative cytokine changes across multiple organs after treatment with lipid nanoparticles (LNPs) or CPC‐derived extracellular vesicles (CPC‐EVs) containing *VEGF‐A* mRNA. Cytokine values are displayed using a signed log10 transformation (sign(x) × log10(|x| + 1)) to (i) accommodate negative values representing decreased cytokine expression relative to PBS controls and (ii) compress the dynamic range while preserving the direction of change (i.e., negative vs. positive values). Each row represents an individual cytokine, and each column represents a specific organ (heart, kidney, liver, lung, pancreas, plasma, spleen and thymus). Colour intensity reflects the relative magnitude of cytokine levels, with stronger colours indicating greater changes in expression. Colour intensity reflects the relative magnitude of cytokine changes. Only cytokines exhibiting significant differences relative to PBS controls are displayed.

Olink‐based cytokine profiling across seven organs and plasma revealed marked differences in immune activation following intravenous delivery of *VEGF‐A* mRNA via LNPs versus CPC‐EVs. Heatmaps illustrate relative cytokine changes across multiple organs after treatment with LNPs or CPC‐EVs containing *VEGF‐A* mRNA. Cytokine values are displayed using a signed log10 transformation (sign(x) × log10(|x| + 1)) to accommodate negative values representing decreased cytokine expression relative to PBS controls, and to compress the dynamic range while preserving the direction of change (i.e., negative vs. positive values) (Figure 5).

LNP‐mediated delivery resulted in significant upregulation of multiple cytokines across several organs, whereas CPC‐EV‐mediated delivery induced fewer cytokine changes and was more restricted and organ‐selective. For LNPs, the highest number of significantly upregulated cytokines was observed in the kidney, followed by plasma and pancreas, with additional cytokine increases observed in the spleen, heart, liver and lung (as summarised in Table 1; Figure S7). In contrast, CPC‐EVs elicited a more restricted cytokine profile and organ‐specific response, with the strongest response observed in the lung. Fewer cytokine responses were observed in the liver, kidney, spleen and plasma. CPC‐EVs induced only one significant cytokine increase in the heart (Table 1; Figure S7).

**TABLE 1:** Olink Target Mouse Cytokine quantification across seven different organs and plasma following intravenous administration of LNPs or CPC‐EVs for *VEGF‐A* mRNA delivery. The table lists cytokines that were significantly upregulated after LNP or CPC‐EV treatment compared to PBS. The values indicate the number of upregulated cytokines. The expression profiles are provided in Figure S7.

	Heart	Liver	Kidney	Pancreas	Spleen	Thymus	Lung	Plasma
LNP‐mediated VEGF‐A mRNA delivery	**4** (CCl12, Ccl2, Ccl4, Il33)	**2** (Ccl12, Ifna2)	**14** (Ccl12, Ccl2, Ccl4, Ccl5, Cd274, Cxcl1, Cxcl9, Ifna2, Ifng, Il1a, Il1b, Il5, Il6, Tnf)	**11** (Ccl2, Ccl22, Ccl4, Ccl5, Cd274, Cxcl1, Cxcl9, Ctla4, Il1a, Il1b, Pdcd1lg2	**5** (Ccl12, Ccl2, Fgf21, Ifna2, Ifnl2)	0	**2** (Ccl2, Cxcl1)	**12** (Ccl12, Ccl4, Ccl2, Cxcl1, Ifna2, Ifnl2, Il10, Il2, Il22, Il4, Il6, Tnf)
CPC‐EV‐mediated VEGF‐A mRNA delivery	**1** (Cxcl9)	**2** (Cxcl9, Pdcd1lg2)	**2** (Ccl5, Il1a)	0	**2** (Csf3, Il27)	0	**18** (Ccl12, Ccl2, Ccl4, Ccl5, Cd274, Csf3, Cxcl1, Cxcl2, Cxcl9, Fgf21, Hgf, Ifng, Il16, Il1b, Il33, Il17, Pdcd1lg2, Tnf)	**2** (Ccl5, Il1b)

Quantitative analysis further showed that LNP‐mediated delivery resulted in increased levels of classical pro‐inflammatory mediators such as Ccl2, Ccl4, Ccl5, Il1a and Il1b, Il6, Tnf and Ifng, with particularly high levels in kidney, pancreas and plasma. In contrast, CPC‐EVs elicited a more selective cytokine profile, characterised by increased levels of Cxcl9, Csf3, Il27, Fgf21, Hgf and Il17a, predominantly in the lung and plasma. Several cytokines, including Ccl12, Ccl2, Ccl4, Cxcl9, Ifng, Il10 and Il33, exhibited elevated levels following delivery by both platforms, although with distinct organ‐specific expression patterns. Direct comparisons between LNPs and CPC‐EVs further demonstrated distinct inflammatory profiles between the two delivery platforms across organs and plasma (Figure S7). The complete expression profile of all cytokines in each organ is presented in Figures S8–S15.

### 2.6 CPC‐EVs Demonstrate Superior Specificity and Fewest Off‐Target Transcriptomic Alterations in Heart Tissue After Local Administration

**FIGURE 6:**
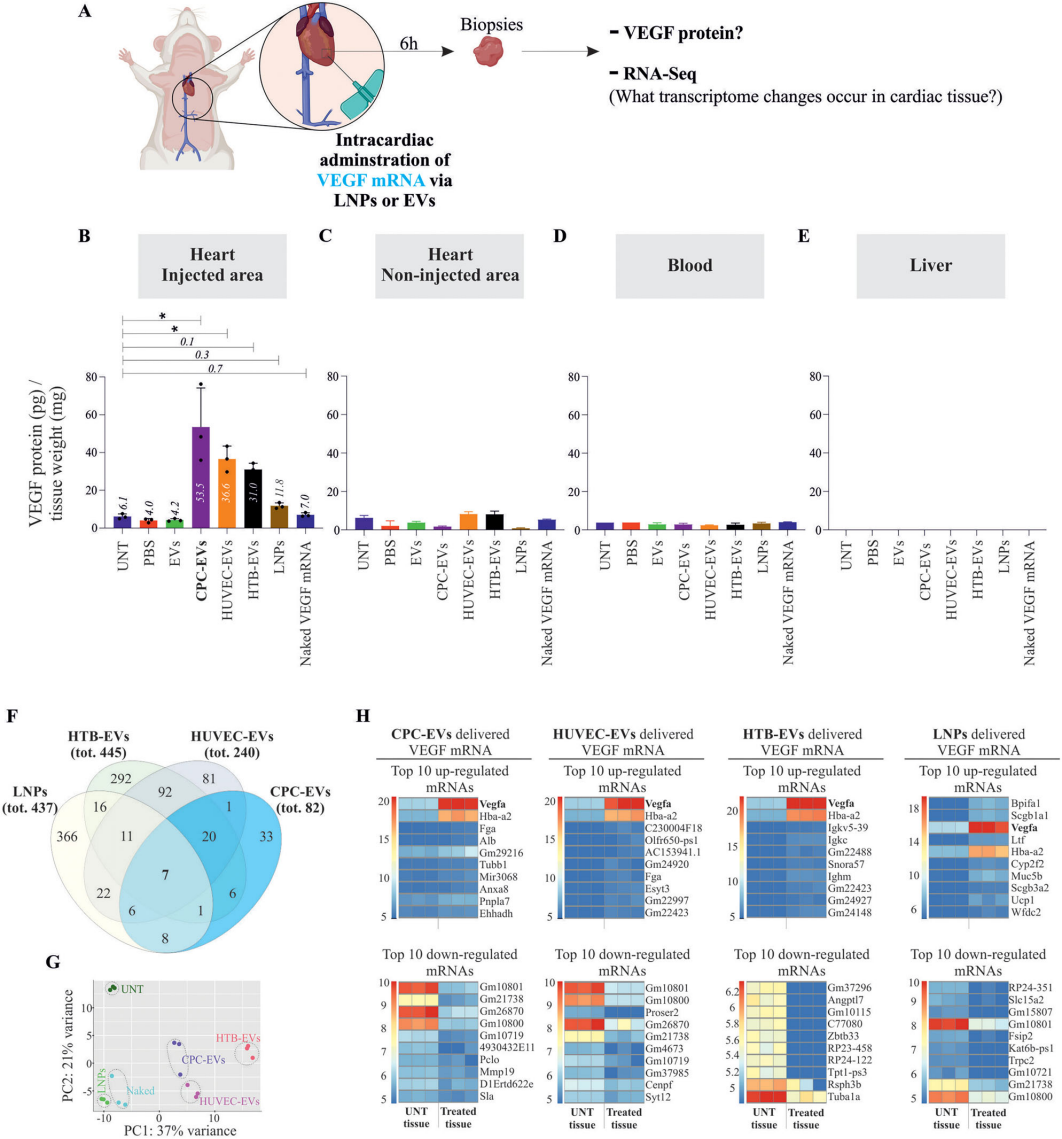
Specificity and off‐target transcriptomic alterations in heart tissue after local administration via CPC‐EVs or LNPs. (A) *VEGF‐A* mRNA was directly injected into a single site of the mouse myocardium using three different EV types (CPC‐EVs, HUVEC‐EVs and HTB‐EVs), LNPs or naked mRNA. At 6 h post‐injection, mice were euthanised, and injected cardiac regions were dissected for analysis of VEGF‐A protein levels and transcriptomic changes. Untreated mice and mice injected with EVs lacking *VEGF‐A* mRNA served as controls. Naked *VEGF‐A* mRNA (without vehicle) was used for comparison with vehicle‐mediated delivery. Injected regions were separated from non‐injected cardiac tissue and analysed for translation of delivered mRNA. (B) Quantification of VEGF‐A protein levels in the injected areas of the heart, following delivery of *VEGF‐A* mRNA via different vehicles. Statistical comparisons among groups were performed using the Kruskal–Wallis test. Statistical significance is indicated as *p < 0.05; whereas exact *p* values are shown for non‐significant comparisons. Data are presented as mean ± SD of *n* = 3 biological replicates per group. (C–E) Evaluation of systemic spillover of *VEGF‐A mRNA* to non‐injected cardiac regions, blood and liver following intramyocardial injection of *VEGF‐A* mRNA via EVs or LNPs. (F) Venn diagram showing the number of gene expression changes in the injected cardiac tissue following *VEGF‐A* mRNA delivery via CPC‐EVs (82), HTB‐EVs (445), HUVEC‐EVs (240) and LNPs (437). CPC‐EVs caused the fewest perturbations in gene expression in the heart, compared with non‐cardiac EVs or LNPs. (G) Principal component analysis (PCA) of transcriptomic profiles in cardiac tissue after injection with *VEGF‐A* loaded EVs, LNPs, or naked mRNA. PCA plots were generated from the top 500 most variable genes. The two principal components (PC1 and PC2) explain 37% and 21% of total variance, respectively, showing clear separation between EV‐based and LNP‐based or naked mRNA‐treated groups. (H) Heatmaps showing the top 10 upregulated and top 10 downregulated transcripts in cardiac tissue after mRNA delivery via EVs or LNPs. The expression intensities are based on variance‐stabilised counts.

To further evaluate the specificity, safety and functional consequences of different mRNA delivery vehicles in cardiac tissue, we directly injected *VEGF‐A* mRNA encapsulated in LNPs, CPC‐EVs, HUVEC‐EVs and HTB‐EVs into a single site of the left ventricular myocardium in mice. The localised delivery specificity of each vehicle was examined by quantifying the resulting protein levels, and off‐target transcriptomic alterations were analysed in the injected areas of heart tissue (Figure 6A).

Equal amounts of *VEGF‐A* mRNA encapsulated in LNPs, or CPC‐EVs, HUVEC‐EVs or HTB‐EVs or in a naked form were injected into a single site of the left ventricular myocardium in mice. The results showed that CPC‐EVs induced higher VEGF‐A protein production among all delivery vehicles, including naked mRNA injection (Figure 6B). No significant increase of the VEGF‐A protein was detected in the proximate non‐injected areas of the heart, the liver or blood (Figure 6C–E), indicating the efficient localised interaction between vehicle (CPC‐EVs) and heart tissue, their subsequent uptake and adaptation.

We next analysed how recipient cells in cardiac tissue responded when mRNA was administered directly to the heart using LNPs and the three EV types. RNA was isolated from the injected area of the heart, and transcriptomic changes were assessed using RNA sequencing. Administration of *VEGF‐A* mRNA via CPC‐EVs caused the fewest changes in the gene expression in cardiac tissue, with only 82 differentially expressed transcripts (Figure 6F). In contrast, mRNA administration via LNPs, HTB‐EVs and HUVEC‐EVs led to significantly higher transcriptomic alterations in the heart tissue with 437, 445 and 240 differentially expressed genes (DEGs), respectively (Figure 6F; Tables S2–S5).

#### 2.6.1 CPC‐EVs Show Distinct Patterns and Narrowest Distribution Dysregulated Genes in heart tissue

Transcripts from the CPC‐EV‐treated group displayed distinct expression patterns across all datasets. Principal component analysis (PCA) of transcriptomic profiles from heart tissue following injection with EVs, LNPs, naked mRNA or no treatment revealed major differences between the groups. Notably, EV‐treated samples separated strongly from the LNP, naked mRNA and untreated groups, accounting for 37% of the total variance (Figure 6G). Collectively, these data demonstrate that delivery via CPC‐EVs not only achieved the highest levels of the VEGF‐A protein but also caused the fewest alterations in cardiac gene expression. This may be due to their tissue‐specific molecular signature that facilitates recognition and uptake by target cells in the heart, thereby improving delivery specificity and efficacy.

Volcano plot analysis further illustrated that CPC‐EVs caused the narrowest distribution of dysregulated genes and were clearly distinct from other vehicle groups (Figure S16A). Comparison of the overlapping gene sets revealed only seven DEGs shared among all four delivery vehicles (Figure 6F; Figure S16B), emphasising the unique and limited perturbative transcriptomic response elicited by CPC‐EVs. Examination of the top 10 upregulated transcripts in heart tissue revealed that when *VEGF‐A* mRNA was delivered via EVs, *VEGF‐A* itself was the most highly represented mRNA (Figure 6H). In contrast, delivery of naked *VEGF‐A* mRNA or encapsulated in LNPs did not result in VEGF‐A being the predominant transcript in cardiac samples. Moreover, CPC‐EVs displayed a distinct pattern among the top 10 downregulated genes following *VEGF‐A* mRNA delivery. The heatmap plots display the top 10 differentially expressed genes in heart tissue (padj ≤ 0.05 and |log_2_FC| > 1). Haemoglobin upregulation may reflect blood contamination rather than true biological regulation.

Together, these findings indicate that CPC‐EVs and LNPs engage in fundamentally different modes of communication with cardiac tissue during mRNA delivery, with CPC‐EVs demonstrating a unique transcriptional response consistent with their cardiac‐specific adaptation and superior delivery efficacy, compared to non‐cardiac EVs or LNPs, or delivery without a vehicle.

#### 2.6.2 Functional Enrichment Analysis of *VEGF‐A* mRNA Delivery via CPC‐EVs Shows Enrichment of Regenerative and Cardiogenic Pathways With Minimal Inflammatory Signalling

Functional enrichment analyses revealed that CPC‐EVs did not induce upregulation of inflammatory genes in the heart, whereas other delivery vehicles, particularly LNPs, elicited a pronounced inflammatory response, with 30 genes upregulated and two downregulated (Table S7). Gene ontology (GO) analysis also showed that among the genes activated following the administration of three EV types or LNPs, only *VEGF‐A* mRNA delivery via CPC‐EVs was associated with the GO term ‘*myocardial cell development*’. This specific response triggers the activation of genes involved in ‘*cardiac muscle cell development*’ and ‘*factors that promote cardiogenesis in vertebrates*’ (Tables S8 and S9). We further identified the top 3 IPA networks in cardiac tissue following *VEGF‐A* mRNA delivery via different vehicles, revealing the key molecular interactions associated with each treatment. For CPC‐EVs, the top networks were primarily associated with cardiovascular disease and organismal injury, immune cell trafficking and cellular movement, and cellular development and proliferation, indicating engagement of regenerative and controlled cellular processes. In contrast, HTB‐EVs were characterised by networks related to metabolic and translational processes, cardiovascular disease and cell death/survival and inflammatory and immunological responses, reflecting broader and more inflammatory biological activation. HUVEC‐EVs exhibited networks associated with vascular and endothelial‐related processes, alongside immune and inflammatory signalling, suggesting an intermediate profile between cardiac‐specific and non‐cardiac EVs. Similarly, LNP‐mediated delivery was predominantly associated with inflammatory and stress‐related networks, including immune activation and cell death/survival pathways, consistent with a more systemic and pro‐inflammatory response (Figures S17–S20). Collectively, these findings indicate that CPC‐EVs uniquely activate regenerative and cardiogenic programs without inducing inflammatory pathways, further highlighting their functional adaptation and safety for cardiac mRNA delivery.

### 2.7 CPC‐EVs Exhibit Enhanced Angiogenic Activity Following *VEGF‐A* mRNA Delivery

**FIGURE 7:**
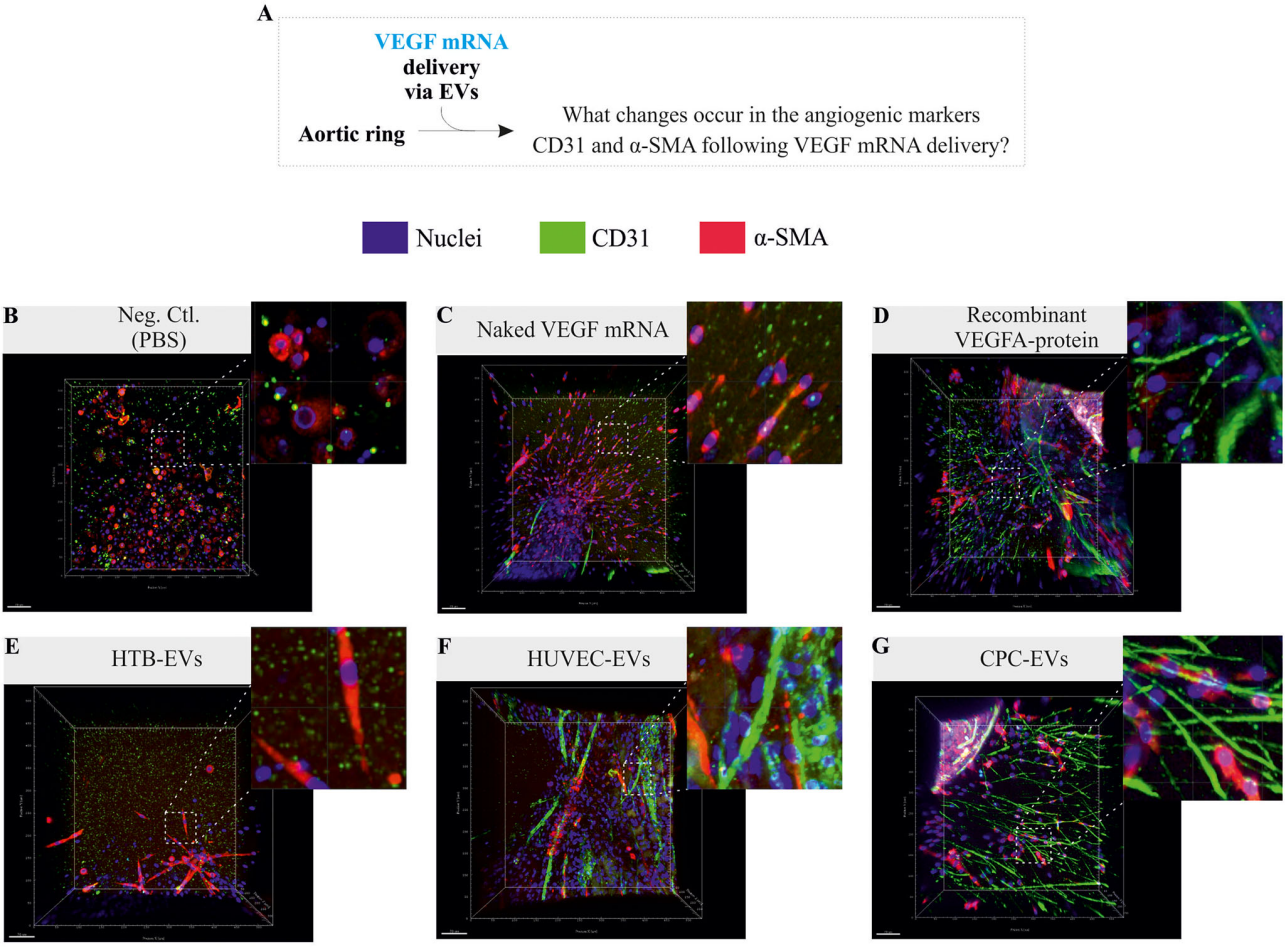
Examination of vessel density by immunostaining for CD31 and α‐SMA expressions using ex vivo aortic ring assay. (A) Schematic representation of the experimental setup to assess vessel density and neoangiogenesis by immunostaining for CD31 and α‐SMA. Aortas from 12‐week‐old male C57BL/6Ncrl mice were collected, sectioned and embedded in growth factor‐reduced Matrigel, followed by treatment with different EV types containing *VEGF‐A* mRNA. Human recombinant VEGF‐A protein was used as positive control, and PBS as negative control. After 12 days, the aortic rings were fixed, immunoassayed for α‐SMA (smooth muscle actin) and CD31 (endothelial marker), and analysed by confocal microscopy. Examination of CD31 and α‐SMA after administration of (B) PBS, (C) naked *VEGF‐A* mRNA, (D) recombinant VEGF‐A protein, or administration of *VEGF‐A* mRNA via (E) HTB‐EVs, (F) HUVEC‐EVs and (G) CPC‐EVs. Neg Ctl, negative control (PBS).

Building on our findings that CPC‐EVs exhibit superior, heart‐targeted mRNA delivery with minimal off‐target distribution and narrowed cytokine induction, we next evaluated the functional consequences of this targeted delivery and compared them with non‐cardiac EVs. Given the challenges associated with assessing angiogenesis in vivo following mRNA administration, we employed an ex vivo aortic ring assay to analyse the angiogenic activity induced by *VEGF‐A mRNA* delivered via cardiac‐specific EVs (CPC‐EVs), and non‐cardiac EVs (Figure 7A). Aortas from 12‐week‐old male C57BL/6Ncrl mice were embedded in growth factor‐reduced Matrigel and treated with *VEGF‐A mRNA*‐loaded EVs. Human recombinant VEGF‐A protein served as a positive control. After 12 days, the aortic rings were fixed and immunostained for α‐SMA and CD31 (markers of microvascular density), and neo‐angiogenesis was analysed by confocal microscopy (Figure 7B–G).

Comparison of CD31 expression revealed that *VEGF‐A mRNA* delivery via CPC‐EVs induced the most extensive vessel outgrowth and the highest microvessel density compared with other EV types (Figure 7G). The quantitative analysis of the aortic ring microscopy images is shown in Figure S21. These results demonstrate that CPC‐EVs not only deliver *VEGF‐A* mRNA efficiently to cardiac tissue but also translate this delivery into a robust angiogenic response ex vivo.

## 3 Discussion

Despite significant progress in mRNA delivery, conventional delivery systems still face major challenges in achieving targeted and efficient transport of mRNA to specific organs or cell types. For therapeutic efficacy, a delivery vehicle must ensure maximal localization of the RNA payload at the intended site of action while minimising systemic exposure, off‐target accumulation and immune activation (Tewabe et al. 2021).

Our study identifies CPC‐derived extracellular vesicles (CPC‐EVs) as an effective vehicle for targeted delivery of exogenous mRNA more efficiently into cardiac tissue. CPC‐EVs displayed pronounced relative cardiac enrichment for mRNA delivery via both systemic and direct intramyocardial injection routes. This achieved both preferential delivery of *VEGF‐A* mRNA and efficient protein translation in the heart while exhibiting the lowest accumulation in the liver. Unlike CPC‐EVs, the LNP‐mRNA accumulated in the liver the most, which is in line with previous studies which have reported that LNPs accumulate in the liver following intravenous administration (Rohner et al. 2022; Paunovska et al. 2019; Kim et al. 2021; Gupta et al. 2021). *VEGF‐A* mRNA levels are reported as nanograms per milligram of tissue for each organ. Normalisation to tissue mass rather than total organ weight was performed to account for substantial inter‐organ size differences, which would otherwise bias absolute mRNA comparisons toward larger organs such as the liver. Reporting mRNA levels per milligram of tissue, therefore, provides a more accurate assessment of relative delivery efficiency and organ‐specific distribution.

Our findings also reveal that intravenous delivery of *VEGF‐A* mRNA via LNPs and CPC‐EVs elicited distinct systemic cytokine responses. LNP‐mediated delivery induced a widespread and robust pro‐inflammatory cytokine activation across several organs and plasma, which suggests that LNPs trigger potent immune activation that may stimulate therapeutic angiogenesis but also increases the risk of systemic toxicity. In contrast, CPC‐EVs elicited a tissue‐selective and narrower cytokine profile. Their distinct organ‐specific profiles, with LNPs acting predominantly in the kidney, pancreas and plasma, and CPC‐EVs in the lung and plasma, highlight differences in biodistribution and immunogenicity. This suggests finely tuned immune modulation, potentially advantageous for improved safety and tolerability, particularly critical for cardiac therapies. It is important to note that cytokine profiling was not extended to HTB‐ and HUVEC‐derived EVs, as they showed limited efficiency in delivering *VEGF‐A* mRNA to the heart and underperformed intramyocardial delivery, compared with CPC‐EVs. Accordingly, our analysis focused on CPC‐EVs, the most effective biological vehicle compared to the standard synthetic counterpart, MC3‐LNPs.

The current study also demonstrated that CPC‐EV‐mediated delivery of *VEGF‐A* mRNA to aortic rings significantly increased microvascular density, as evidenced by enhanced CD31 and α‐SMA immunofluorescence staining. This enhanced angiogenic response suggests the therapeutic potential of *VEGF‐A* mRNA in promoting neovascularisation. Administration of *VEGF‐A* mRNA to heart tissue is thought to be a promising approach for the treatment of cardiovascular diseases, by stimulating neovascularisation (angiogenesis) (Anttila et al. 2020; Collen et al. 2022).

The use of CPC‐EVs with their inherent tropism for cardiac tissue is especially advantageous for transporting *VEGF‐A mRNA* to the heart and achieving maximal protein expression for neovascularisation. Even if it is a fraction of the delivered *VEGF‐A* mRNA that reaches the heart via non‐invasive routes, this would represent a significant advantage for treating ischaemic cardiovascular diseases. However, future studies using in vivo ischaemia models, such as hindlimb ischaemia, could further validate the therapeutic efficacy of EV‐mediated *VEGF‐A* mRNA delivery. Therapeutic vascularisation with VEGF‐A protein has been studied for decades experimentally and clinically (Gaffney et al. 2007; Giacca and Zacchigna 2012; Yla‐Herttuala 2013). In this context, our findings support the concept that targeted mRNA delivery can effectively stimulate vascular growth in a controlled manner. Targeting the heart via EVs, therefore, could benefit patients for which no delivery vehicles are currently available to deliver *VEGF‐A* mRNA in the heart.

Studies have demonstrated robust, yet transient, expression of VEGF‐A protein for several days using chemically modified *VEGF‐A* mRNA in mouse models, large animals and even in a first‐in‐human clinical study (Collen et al. 2022; Zangi et al. 2013; Carlsson et al. 2018; Gan et al. 2019; Sun et al. 2018; Pehrsson et al. 2019; Chien et al. 2014). In Phase IIa clinical trials involving cardiac patients undergoing open‐heart surgery, the naked form of *VEGF‐A* mRNA without a delivery vehicle was directly injected into hypoperfused regions of the heart (Anttila et al. 2020; Collen et al. 2022; Anttila et al. 2023). LNP‐mediated delivery of eGFP mRNA has been shown to be more efficient than naked mRNA in a citrate buffer upon direct intramyocardial injection in mice (Labonia et al. 2024). However, recognising the limitations of invasive local myocardial injection of naked mRNA into tissues, systemic or minimally invasive delivery strategies that achieve cardiac enrichment are critical for translating *VEGF‐A* mRNA therapy beyond surgical settings. Instead of intramyocardial injections, the mRNA embedded in nanocarriers, such as EVs derived from cardiac cells, can be employed as a non‐invasive systemic delivery of mRNA to target the heart. Therefore, the present study examined the specificity, delivery efficiency and potential off‐target effects of LNPs and three distinct biological vehicles when used for direct injection of *VEGF‐A* mRNA into the heart, compared to i.v. administration.

Compared to LNPs, which do not express cell‐derived surface proteins, the enhanced cardiac specificity of CPC‐EVs could be attributed to their surface proteins being recognised by cardiac homing mechanisms, thereby promoting preferential accumulation in the heart. This is consistent with previous reports that EV biodistribution is determined by their cell of origin and targeting properties (Wiklander et al. 2015). It has also been suggested that EVs or biomimetic nanoparticles can retain the surface characteristics of their parental cells, thereby conferring intrinsic tissue‐specific targeting capabilities (Tian et al. 2021). To maximise mRNA delivery to heart, CPC‐EVs can be engineered through surface modifications such as conjugation with targeting ligands, thereby improving organ‐ or cell‐specific recognition. Future studies should identify the molecular determinants responsible for cardiac enrichment.

The current study also has several limitations; first, although the study demonstrates preferential enrichment of *VEGF‐A* mRNA and protein at the organ level in the heart, it does not define the specific cardiac cell populations within the myocardium responsible for mRNA uptake in vivo. It is plausible that cardiac endothelial cells, which form the luminal interface and are directly exposed to circulating EVs, represent primary recipients of CPC‐EV–delivered mRNA. Such an uptake would be consistent with the observed angiogenic response. However, definitive identification of recipient cell types will require cell‐type‐resolved approaches such as co‐localisation studies, in situ hybridisation or single‐cell transcriptomic analysis. This will further clarify intra‐cardiac distribution and targeting mechanisms. Secondly, the small sample size (*n* = 3) in the in vivo biodistribution experiments limits statistical power. While non‐parametric analyses (Kruskal–Wallis test) were appropriate for datasets with small sample sizes, the limited number of biological replicates may reduce the ability to detect biologically relevant differences. Therefore, these findings should be interpreted as indicative rather than definitive. However, our conclusions are based on consistent distribution patterns observed across multiple organs and supported by complementary readouts, including mRNA levels, protein expression and heart‐to‐organ ratios. Larger sample sizes would further enable more precise estimation of effect sizes. Accordingly, our findings should be interpreted within the context of comparative and mechanistic investigation rather than as definitive preclinical efficacy conclusions. Additionally, in our study, CD63^+^ CPC‐EVs showed minimal CD9 expression. EV marker expression varies by parental cell type and EV subtype. This could possibly explain the limited CD9 signal, as CD9 is not uniformly expressed across all EV populations, and CD63^+^ EVs may lack CD9 (Nawaz et al. 2023; White et al. 2022). Accordingly, the CD63^+^/CD9^−^ EV ratio increases due to a relative enrichment of CD63^+^ EVs and reduced CD9 expression (Duke et al. 2025). Consistent with this, in our immunocapture/FACS workflow, CPC‐derived EVs were robustly captured via CD63 but exhibited minimal CD9 co‐positivity, suggesting enrichment of a CD63‐dominant subtype. CPC‐EV identity is supported by orthogonal characterisation, including SEC‐based isolation, TEM morphology and NTA size distribution.

## 4 Methods

### 4.1 Construction of *VEGF‐A* mRNA Sequence and Clean Capping

The CDS sequence of *VEGF‐A* 165 (isoform 11) containing an open reading frame with start and stop codons (576 nucleotides including atg start and tga stop codons, encoding 191 amino acids of VEGF‐A protein) was selected. The sequence code was provided to TriLink Biotechnologies (CA, USA) for the *VEGF‐A* mRNA construct with clean cap modifications. A CleanCap (AG, polyadenylated) method was applied, which is a co‐transcriptional capping method with a fully processed mature mRNA and was optimised for mammalian systems. After clean cap modifications with polyadenylation, the resulting mRNA length was 852 nucleotides. The prepared *VEGF‐A* mRNA was dissolved in 1 mM sodium citrate buffer (pH 6.4), and stored at –80°C. The sense strand of *VEGF‐A* mRNA used in this study is presented in Figure S1B, and the sequence of the encoded VEGF‐A protein is provided in Figure S1C. The VEGF‐A protein encoded from the given sense strand of *VEGF‐A* mRNA was obtained using the transcription and translation tool http://biomodel.uah.es/en/lab/cybertory/analysis/trans.htm.

### 4.2 mRNA Loading and Characterisation of Formulated LNPs

DLin‐MC3‐DMA LNPs containing modified *VEGF‐A* mRNA (852 nucleotides, 5meC, Ψ; TriLink Biotechnologies, USA) were prepared by precipitating the mRNA with four different lipid components as described previously (Nawaz et al. 2023). These components consist of an ionisable lipid, DLin‐MC3‐DMA (MC3), two helper lipids (DSPC and Cholesterol) and a PEGylated lipid (DMPE‐PEG2000). The chemical structure of MC3‐LNPs is presented in Figure S1A. A solution of *VEGF‐A* mRNA in water was prepared by mixing mRNA dissolved in MilliQ‐water, 100 mM citrate buffer pH = 3 and MilliQ‐water to give a solution of 50 mM citrate. Lipid solutions in ethanol (99.5%) were prepared with a composition of four lipid components [MC3:Cholesterol:DSPC:DMPE‐PEG2000] = 50:38.5:10:1.5 mol% and a total lipid content of 12.5 mM. The mRNA and lipid solutions were mixed in a NanoAssemblr (Precision NanoSystems, Inc., BC, Canada) microfluidic mixing system at a volume mixing ratio of 3:1 and a constant total flow rate of 12 mL/min. At the time of mixing, the ratio between the nitrogen atoms on the ionisable lipid and phosphor atoms on the mRNA chain was 3.08:1. In some preparations of LNPs, CleanCap Cy5‐*eGFP* mRNA (996 nucleotides, 5meC, Ψ) and CleanCap *eGFP* mRNA (Trilink Biotechnology) were mixed in a 1:1 ratio and encapsulated instead of *VEGF‐A* mRNA. The initial 0.25 mL and the last 0.05 mL of the LNP solution prepared were discarded, while the rest of the volume was collected as the sample fraction. The sample fraction was transferred immediately to a Slide‐a‐lyzer G2 dialysis cassette (10000 MWCO, Thermo Fisher Scientific Inc.) and dialysed overnight at 4°C against PBS (pH 7.4) to remove residual ethanol (25% v/v). The volume of the PBS buffer was 600× the sample fraction volume. The dialysed sample was collected and filtered through a 0.22 µm sterile filter (Gillex, Merck) prior to any characterisation.

### 4.3 Culturing and Treatment of HTB‐177, HUVECs and CPCs With LNP‐*VEGF‐A* mRNA

The human epithelial HTB‐177 (NCI‐H460, ATCC) cell line was cultured according to the manufacturer's protocol. The cells were cultured in RPMI‐1640 growth medium containing sodium bicarbonate, without sodium pyruvate and HEPES (Sigma–Aldrich), supplemented with 10% EV‐depleted Fetal bovine serum (FBS) (Sigma–Aldrich), 1% of L‐glutamine and 1% penicillin‐streptomycin (Thermo Fisher Scientific), at 37°C and 5% CO_2_. The medium was replaced with a fresh medium after 48 h, followed by adding LNP‐*VEGF‐A* mRNA to the cells in culture for an experimental period of 24 h. The heat‐inactivated FBS was prepared by incubating it at 56°C for 1 h. EV‐depletion was achieved by ultracentrifugation at 120,000 × *g* for 2 h at 4°C using an Optima L‐100 XP ultracentrifuge with a 70Ti rotor (Beckman Coulter). The EV‐depleted supernatant was then filtered through 0.2 µm filters before being incorporated into the RPMI‐1640 growth medium. The HUVECs cell (Lonza, Switzerland), were plated and expanded in culture medium according to the manufacturer's instructions (CC‐5035 EGM‐PLUS BulletKit Medium; CC‐5036 EGM‐PLUS Basal Media + CC‐4542 EGM‐PLUS SingleQuots Kit, Lonza, Switzerland). Briefly, cells were cultured in T‐75 cm (Tewabe et al. 2021) culture flasks with EGM‐Plus medium and incubated at 37°C in 5% CO_2_, and 95% saturated atmospheric humidity. The culture medium was replaced with fresh media every 2 days until the cells attained around 80% confluency, and then the cells were expanded. At 80% confluency, the cells were rinsed with Ca^++^/Mg^++^ free Dulbecco's phosphate‐buffered saline. TrypLE Express Enzyme (1X), without phenol red, was added to detach the cell layer from the flask. The enzyme activity was stopped by adding the complete culture medium to the flask. The cells were aspirated by gently pipetting and transferred to a tube and centrifuged at 1000 rpm for 5 min. The HUVEC cell pellet was resuspended with fresh culture medium and dispensed into a T‐75 cm (Tewabe et al. 2021) culture flask at a density of 2 × 10^6^ cells/flask and incubated at 37°C and 5% CO_2_ for 24 h, followed by the addition of LNP‐*VEGF‐A* mRNA to each flask for an experimental period of additional 24 h. At the endpoint, the conditioned medium was collected for EV isolation. Also, the cells were detached from the culture flask using TrypLE, as described before. The cells were counted and checked for their viability before centrifugation. The cell pellets were used for further analysis. All the steps were carried out under aseptic conditions.

iCell cardiac progenitor cells (R1093, Fujifilm Cellular Dynamics, Madison, WI, USA) were thawed, centrifuged (180 × *g* for 5 min), resuspended in the maintenance medium composed of William's E Medium, Cocktail B (Thermo Fisher Scientific) and seeded in fibronectin‐coated (1 mg/mL fibronectin solution diluted in sterile D‐PBS to a final concentration of 5 µg/mL immediately before use, Sigma–Aldrich, St. Louis, MO, USA) 6‐well plates at a density of 5 × 10^5^ cells/well. Cells were then incubated at 37°C in the ambient atmosphere supplemented with 5% CO_2_ and 95% relative humidity. The medium was replaced with a fresh maintenance medium after 24 h, followed by adding LNP‐*VEGF‐A* mRNA to each well for an experimental period of an additional 24 h. At the endpoint, the conditioned medium was collected for EV isolation. Also, the cells were detached from the culture plates using TrypLE, as described before, counted and checked for their viability. The pelleted cells were used for further analysis. All the steps were carried out under aseptic conditions.

### 4.4 Characterisation of the Formulated LNPs

The intensity‐averaged particle size (Z‐average, d_Z_) was measured using ZetaSizer (Malvern Instruments Inc.). The measurement solution was made by diluting 20 µL of the sample fraction using 980 µL PBS (pH 7.4). The mRNA concentration and encapsulation efficiency (EE) of the final product was measured by Quant‐it Ribogreen Assay Kit (Thermo Fisher Scientific).

### 4.5 Isolation and Separation of Extracellular Vesicles From LNP‐Treated Cells

Prior to EV isolation, the cell debris was removed from the collected conditioned medium by centrifugation at 3000 × *g* for 15 min at 4°C on a 4K15 centrifuge (Sigma–Aldrich). EVs from the conditioned media of LNP‐mRNA‐treated cells and controls were isolated by size exclusion chromatography (SEC) using qEV‐70/10 mL columns (Izon Science Ltd, New Zealand) according to the manufacturer's guidelines, which suggest the collection of four fractions, 5 mL each. However, some modifications were applied to the original protocol, with the following amendments. (i) 15 mL of media was used instead of 10 mL of media. (ii) As we had 15 mL of media, instead of four fractions, six fractions (5 mL each) that typically represent EV fractions were collected for EV analysis. Each fraction was concentrated using 30 KDa Amicon Ultra/15 mL Centrifuge Filters (cat: # UFC903024, Sigma–Aldrich, now Merck), at 4000 x *g*, for 25 min, 4°C. The total RNA was isolated from each fraction, and *VEGF‐A* mRNA was detected/quantified by qPCR, in each fraction. Fractions 1–6 were analysed for the presence of *VEGF‐A* mRNA and protein. (iii) Fractions 7–12 (non‐EV fractions) were also collected to analyse any mRNA. The VEGF‐A protein was analysed in all fractions, 1–12 (15 mL from T75 flasks HTB‐177 cultures, and from six‐well plates of CPC cultures: 5 wells × 3 mL = 15 mL). Briefly, after loading 15 mL of sample into the column reservoir, a 20 mL void volume was first discarded, followed by the collection of 12 fractions, each of a 5 mL collection (elution) volume.

### 4.6 Quantification of *VEGF‐A* mRNA in SECEV Fractions

The total RNA from LNP‐mRNA treated HTB‐177, HUVECs, CPCs and their secreted EVs was isolated using miRNeasy Mini Kit (Qiagen, cat. #: 217004) according to the manufacturer's guidelines. Total RNA was quantified by Qubit 2.0 fluorometer (Thermo Fisher Scientific) and NanoDrop 1000 (Thermo Fisher Scientific). The RNA quality was assessed by a 230/260 ratio recorded on NanoDrop. RNA samples from untreated cells and their EVs were used as controls.

LNP‐*VEGF‐A* mRNA from cell lysates and their secreted EVs was detected and quantified by real‐time qPCR. 30–50 ng of total cellular total RNA were reverse transcribed into cDNA using a high‐capacity cDNA kit with RNase inhibitor (Thermo Fisher Scientific: 4374966). 45 ng of cDNA was used for *VEGF‐A* mRNA quantification using hydrolysis probes (TaqMan probe assay, Thermo Fisher Scientific, assay ID: Hs00900055_m1) on ViiA 7 instrument according to the manufacturer's instructions. To generate the standard curve for absolute quantification, the *VEGF‐A* mRNA standards were prepared using pure *VEGF‐A* mRNA (TriLink Biotechnologies, USA). 2 µg of the pure *VEGF‐A* mRNA was reverse transcribed into cDNA, and *VEGF‐A* cDNA was serially diluted 10‐fold (highest point: 100 ng, and lowest point: 0.0001 ng) to generate a standard curve. The assay was performed in technical triplicates. For the absolute quantification of *VEGF‐A* mRNA, the cDNA from the cell and EV samples was interpolated against the *VEGF‐A* standard curve with minimal R^2^ > 0.975. GAPDH gene (Thermo Fisher Scientific, assay ID: Hs02758991_g1) was used as an internal control.

### 4.7 Detection of VEGF‐A Protein in SEC Fractions

The VEGF‐A protein was quantified in the individual SEC fractions (F1‐12) isolated from HTB‐177 treated with LNP‐*VEGF‐A* mRNA. EV fractions isolated from untreated cells were used as controls. The human VEGF‐A sandwich ELISA Kit (cat.#: RAB0507, Sigma–Aldrich, now Merck) was used to detect and quantify the VEGF‐A protein according to manufacturer's instructions. 100 µL of EV solution or serially diluted VEGF‐A protein standards were added per well. VEGF‐A protein concentration (pg/mL) was recorded on ELISA reader instrument (Spectra max, 340 PC, molecular devices), as the VEGF‐A protein levels relative to VEGF‐A standard curve. The levels of VEGF‐A protein in EVs were normalised to total EV‐proteins (µg).

### 4.8 Characterisation of Extracellular Vesicles Isolated by Size Exclusion Chromatography

#### 4.8.1 Determination of Size and Concentration of SEC‐EVs

The size and concentration (particle number) of EVs isolated by size exclusion chromatography from all three cell types were assessed using Nanoparticle Tracking Analysis (NTA) with an LM14c instrument from Malvern Panalytical, United Kingdom, equipped with an sCMOS camera.

Initially, the EV pellets were dissolved in 1000 µL of PBS and then further diluted 5‐fold with PBS to ensure that the number of particles in the field of view remained below 100 particles per frame. Independent measurements, in scatter mode, were performed on two biological replicates from each time point. Each EV sample was analysed through three captures, with a total of 2248 frames, using an adjusted camera level of 16 and a detection threshold of 5. The settings for Blur and Max Jump Distance were left on auto. Data acquisition and analysis were carried out using NanoSight Fluorescent NTA LM14c software version 3.2 (Malvern Panalytical, UK).

#### 4.8.2 Detection of CD63 and CD9 EV Markers in SEC‐EVs

EVs were initially isolated using size exclusion chromatography as described above. Subsequently, the CD63 and CD9 positive EVs were isolated using an immunoaffinity‐based method. The CD63 isolation/detection reagent for cell culture medium (Thermo Fisher Scientific, cat.#: 10606D) was used to immobilise the CD63^+^ EVs to magnetic dynabeads conjugated with anti‐CD63 antibody. In the binding reaction, 30 µL of CD63‐antibody conjugated beads were incubated with 60 µg of EVs (beads + EVs; total volume 120 µL). As a negative control, 30 µL of CD63‐antibody conjugated beads alone were incubated with an equivalent volume of PBS (no EVs). The EVs were immobilised on anti‐CD63 beads and incubated at 4°C overnight (Day 1). Then the next day (Day 2), unbound beads or EVs were washed four times with BSA‐PBS isolation buffer (0.25% BSA dissolved in PBS). The washing steps were performed using magnetic separators (EasySep, StemCell technologies), according to the manufacturer's protocol (cat.#: 10606D). After the final wash, the immobilised CD63^+^ EVs were suspended in 120 µL of BSA‐PBS isolation buffer and then further stained with a mouse anti‐human PE‐CD9 antibody (BD Pharmingen, cat.#: 555372). A 20 µL of PE‐CD9 antibody was added to the CD63^+^ EV solution and incubated in a sample shaker for 1 h, at room temperature (in the dark). To remove unbound CD9‐antibody, the sample was washed four times with BSA‐PBS isolation buffer using magnetic separators and finally suspended in 200 µL of isolation buffer. The immobilised CD63^+^ EVs were acquired on a BD FACSLyric system (BD Biosciences) to detect CD9^+^ EVs. The data were analysed using FlowJo software (TreeStar Inc.). The experiment was performed in biological duplicates.

#### 4.8.3 Transmission Electron Microscopy (TEM) Analysis of SEC‐EVs

The SEC‐EVs isolated from HTB‐177, HUVECs, and CPCs were fixed in 2% Paraformaldehyde–0.1 M phosphate‐buffered saline for 30 min. Subsequently, a Glow discharge technique (30 s, 7,2 V, using a Bal‐Tec MED 020 Coating System) was applied over carbon‐coated copper grids, and immediately, the grids were placed on top of sample drops for 15 min. Then, the grids with adherent EVs were washed in a 0.1 M PBS drop. An additional fixation in 1% glutaraldehyde was performed for 5 min. After washing properly in distilled water, the grids were contrasted with 1% uranyl acetate and embedded in methylcellulose. Excess fluid was removed and allowed to dry before examination with a transmission electron microscope FEI Tecnai G2 Spirit (Thermo Fisher Scientific, Oregon, USA). All images were acquired using Radius software (Version 2.1) with a Xarosa digital camera (EMSIS GmbH, Münster, Germany).

#### 4.8.4 EV‐Mediated Delivery of Translatable *VEGF‐A* mRNA to Human Endothelial Cells In Vitro

Further, we investigated whether EVs isolated by SEC could deliver a *VEGF‐A* mRNA to cells. Pooled and concentrated EV fractions (F2‐F6) of HTB‐EVs, HUVEC‐EVs and CPC‐EVs containing *VEGF‐A* mRNA (550 ng), were delivered to HUVECs. LNP‐*VEGF‐A* mRNA was used as a positive control. Untreated cells and EVs without *VEGF‐A* mRNA were used as negative controls. 24 h post‐incubation, the production of VEGF‐A protein was quantified in the conditioned medium using the Human VEGF‐A sandwich ELISA Kit. The presence or absence of endogenous *VEGF‐A* mRNA was also investigated in untreated samples.

#### 4.8.5 Intramuscular and Intravenous Delivery of Luciferase mRNA via SEC‐EVs or LNPs and IVIS Analysis

SEC‐EVs or LNPs containing firefly luciferase mRNA (FLuc‐mRNA, 215 ng, in 30 µL) were administered intramuscularly to female C57bl/Ncr mice. In a separate experiment, mice were intravenously administered with EVs or LNPs containing FLuc‐mRNA (1 µg, in 100 µL). To confirm the translation of mRNA into luciferase, a luciferin (5 mL/kg) was administered intramuscularly or intravenously 6 h after the FLuc‐mRNA delivery. The mice were terminated 20 min after the luciferin administration, and the organs were dissected and scanned with an IVIS Spectrum within 5 min of termination. The total radiance was quantified as a factor of luciferase produced from FLuc‐mRNA. After intramuscular delivery of FLuc‐mRNA, the radiance (luciferase/luciferin activity) was quantified in dorsal and left muscle of mice after Fluc‐mRNA delivery via HTB‐EVs, and LNPs.

#### 4.8.6 Biodistribution of *VEGF‐A* mRNA Delivered via EVs or LNPs

Female C57bl/Ncr mice were intravenously administered with 100 µL of EVs or LNPs containing 1 µg of *VEGF‐A* mRNA. Untreated mice (PBS) were used as controls. The mice were sacrificed 6 h post administration, and organs were collected and snap frozen. Total RNA and total proteins were extracted from the organs as described above. qPCR was performed to quantify the *VEGF‐A* mRNA as described above. VEGF‐A protein was quantified using the human VEGF‐A sandwich ELISA Kit (cat.#: RAB0507, Sigma–Aldrich, now Merck) according to the manufacturer's instructions. *VEGF‐A* mRNA and protein levels were normalised to tissue mass and are reported as ng/mg tissue, corresponding to values presented in the figures as “ng per weight of tissue (mg).”

#### 4.8.7 Intramyocardial Injections of *VEGF‐A* mRNA via EVs or LNPs and Detection of VEGF‐A Protein

The animal experiments followed the NIH guidelines and were approved by the Gothenburg University Animal Ethics Committee (Gothenburg Ethical Review Board number Ea 001173–2017). Male C57BL/6Ncrl mice at 10–12 weeks of age and weight of ∼25 g, were purchased from Charles River and housed on a 12 h light/12 h dark cycle, ambient temperature at 21°C–22°C and 50% humidity. On the day of the injections, mice were anaesthetised with 2%–3% Isoflurane mixed with oxygen, intubated and connected to a ventilator. The mice were ventilated with air ∼800 mL/min and oxygen ∼100 mL/min (∼230 strokes/min; MiniVent Ventilator for Mice (Model 845), Harvard Apparatus, Holliston, MA). Core temperature was continuously monitored and maintained at 35°C–36.5°C by a heating operating table and heating lamp controlled by a rectal thermometer. Electrodes were inserted under the skin to register the heart rate and electrical activity (PharmLab, Paris, France). The mice were subjected to a left thoracotomy at the fourth intercostal space, ∼2–3 mm to the left of the sternum. A rib spreader was used to keep the incision open. The pericardium was opened, and the heart was held using a USP 8‐0 suture (Braun, Kronberg im Taunus, Germany) and 40 µL of EVs or LNPs (corresponding to 50 ng *VEGF‐A* mRNA) or PBS (no mRNA) treatments were injected in one single site in the myocardium of the left ventricle using an insulin syringe (Becton, Dickinson and Company, Franklin Lakes, NJ). 50 ng of naked *VEGF‐A* mRNA in citrate saline solution (without loading into EVs or LNPs) was also injected into separate groups. The chest was then closed, and the mice were monitored during continued maintenance of body temperature and ventilation until they regained consciousness and could be disconnected. The mice were sacrificed 6 h post‐injections, and the heart, liver and blood were collected. For the heart, the area of injection in the left ventricle was dissected and separated from the rest of the heart and snap‐frozen. The remaining parts of the heart (remote left ventricle, right ventricle and atria) were snap‐frozen in a second tube and referred to as a remote non‐injected area for further analysis. The animals were divided into five groups, either for vehicles loaded with *VEGF‐A* mRNA such as HTB‐EVs, HUVEC‐EVs, CPC‐EVs, LNPs or naked *VEGF‐A* mRNA in a citrate buffer without a vehicle. Each group consisted of three individuals, and they were injected separately. Untreated naïve mice or those treated with an equal volume of PBS with mRNA were used as controls.

#### 4.8.8 Quantification of Human *VEGF‐A* mRNA in tissue samples

At 6 h post‐injection of *VEGF‐A* mRNA via SEC‐EVs, the total mRNA and total proteins from injected and non‐injected heart areas, as well as from the liver were extracted. Total RNA from 20 to 70 mg of each tissue was extracted using miRNeasy Mini Kit (Qiagen, cat. #: 217004). The tissue was placed in 2 mL Eppendorf tubes with 700 µL of QIAzol lysis reagent and beads and were lysed in Tissue LyserII (Qiagen) for 5 min at the maximum speed (30 Hz). The RNA isolation steps were followed according to the manufacturer's guidelines provided with kit. Total extracted RNA was quantified by Qubit 2.0 Fluorometer (Thermo Fisher Scientific) and NanoDrop 1000 (Thermo Fisher Scientific). The RNA quality was assessed by a 230/260 ratio recorded on NanoDrop. RNA samples from untreated cells and their EVs were used as controls. *VEGF‐A* mRNA was quantified in each sample using qPCR, as described above.

#### 4.8.9 Quantification of VEGF‐A Protein in Tissues and Plasma

Total protein from the tissues, including injected and non‐injected areas of the heart as well as liver, lung, pancreas, kidney, spleen and thymus was extracted using T‐PER Tissue Protein Extraction Reagent (Thermo Fisher Scientific Cat. 78510) in the presence of 1% halt protease inhibitor cocktail, EDTA free (Thermo Fisher Scientific, cat.#: 87785), following the manufacturer's instructions. Briefly, 20–70 mg of tissue was lysed in 250 µL of lysis reagent supplemented with protease inhibitors and beads using TissueLyser II (Qiagen) at maximum speed (30 Hz) for 5 min. Tissue lysates were centrifuged at 10,000 × *g* for 15 min at 4°C, and the supernatant was collected while the pellet was discarded. The blood samples were centrifuged at 2000 × *g* for 5 min at 4°C to obtain plasma. The protein concentrations were quantified using Qubit 2.0 Fluorometer (Thermo Fisher Scientific).

Human VEGF‐A protein was quantified by VEGF‐A sandwich ELISA (Cat. #: RAB0507, Sigma–Aldrich) performed separately on the samples from the injected area of the left ventricle and proximate non‐injected areas of the heart, as well as the liver, and blood. The amount of VEGF‐A protein (pg/mL) in each organ was normalised to the relative organ weight (g).

#### 4.8.10 Multiplex Cytokine Profiling in the Tissues and Plasma

Total protein was extracted from tissues and plasma as mentioned above. Cytokine profiling was performed across seven organs and plasma to capture both tissue‐specific and systemic responses. Expression of 48 cytokines was quantified using the Olink Target 48 Mouse Cytokine panel, based on Proximity Extension Assay (PEA) technology. Samples from seven tissue types (heart, lung, liver, spleen, thymus, kidney and pancreas) and plasma were submitted to Olink Proteomics (Uppsala, Sweden) for analysis. Equal amounts of total protein (40 µL of sample containing 0.5 mg/mL) from each organ and plasma were subjected to cytokine profiling. Protein abundance was reported both as Normalised Protein eXpression (NPX) values (log2 scale, relative quantification) and as absolute concentrations (pg/mL) where available. Internal and external controls provided by Olink were included for quality control and normalisation, following the manufacturer's standard data processing pipeline.

#### 4.8.11 Aortic Ring Assay

The aortic ring assay was performed as described in Baker et al. (2011). Aortas from 12‐week‐old male C57BL/6Ncrl mice were collected. Fat tissue was removed from the aorta using forceps and blood remaining inside was washed out by flushing Opti‐MEM (ThermoFisher Scientific) supplemented with 2.5% of Fetal Bovine Serum (FBS; ThermoFisher Scientific) and 1% of Penicilin‐Streptomycin (P/S; ThermoFisher Scientific). Then, the aorta was sliced into 0.5 mm rings and serum starved overnight in Opti‐MEM supplemented with 1% P/S. Rings from different aortas were kept separated. The following day, aortic rings from different animals were randomised and embedded individually in 50 µL of growth factor‐reduced Matrigel (Corning) on 96‐well plates. After embedding aortic rings, Opti‐MEM medium supplemented with 2.5% FBS and 1% P/S, with the different treatments added to every well. A concentration of 10 µg/mL of SEC‐EV total protein containing *VEGF‐A* mRNA was used for treatments. Human Recombinant VEGF‐A protein at 20 ng/mL was used as positive control, and PBS was used as negative control. Maintenance media and treatments were refreshed every other day for 12 days. After 12 days, aortic rings were fixed and stained for α‐SMA (Dako, M0851) and CD31 (R&D Systems, AF3628) as described in Baker et al. (2011).

#### 4.8.12 Confocal Images Acquisition

The confocal images of the aortic rings stored in Falcon 96‐well plate format were captured on an inverted Zeiss LSM880 confocal microscope equipped with Airyscan detector using Plan‐Apochromat 20×/0.8 M27 air objective lens. One field of view image was acquired from each well and performed in triplicate with a total of three different wells per biological condition. DAPI, CD31 and α smooth muscle actin (α SMA) were imaged using 405, 488, and 633 nm excitation lasers, respectively. The scanning acquisition parameters are 16‐bit depth, 532 µm by 532 µm x‐by‐y frame resolution and 8 µm z‐stack thickness. The total scanning thickness is ∼250 µm.

#### 4.8.13 3D Volumetric Projection Images

For presentation and qualitative display, the .czi file images obtained from Zeiss LSM880 confocal microscope were extracted and 3D‐projected with Imaris version 9.0.1 (Oxford Instruments, Bitplane AG, Switzerland).

#### 4.8.14 Image‐Based Analysis of Vascular Network Architecture

The quantitative analysis of the images to determine the total branches, total junctions and branch average lengths was performed with skeleton analysis in ImageJ version 1.53f51 (National Institute of Health, USA). Firstly, the multichannel.czi images were splitted into each channel. The analysis was focused only on the 488 nm/green channel (CD31) and 633 nm/far red channel (α SMA). These images were then converted into 8‐bit images, morphology‐processed into 2‐pixel circles open, and then stacked as z‐projection sum slices. The skeletonised plugin was then chosen with the lowest intensity voxel; no prune ends elimination, largest shortest path calculated and labelled skeletons displayed. The final numerical data was eventually obtained as .csv files.

#### 4.8.15 Quantitative Analysis of Vascular Network Formation

Quantitative analysis of the aortic ring microscopy images was performed using ImageJ, a Java‐based image processing program developed by the National Institutes of Health (NIH) and the Laboratory for Optical and Computational Instrumentation (LOCI, University of Wisconsin).

#### 4.8.16 Transcriptomic Analysis of Mouse‐Heart Tissue After Direct Injection of *VEGF‐A* mRNA via EVs or LNPs

The transcriptomic analysis of mouse heart tissue after direct injection of *VEGF‐A* mRNA via EVs or LNPs involved obtaining heart tissue biopsies following injection of *VEGF‐A* mRNA via HTB‐EVs, CPC‐EVs, HUVEC‐EVs or LNPs, with untreated mice as controls. Total RNA was *isolated*, and RNA‐seq was performed using NovaSeq6000. The DESeq2 R‐package was utilised for differential expression analysis, and explorative data analysis included PCA, Volcano plots and Heatmap plots. Venn diagrams were employed to compare DEGs, and functional analysis was conducted on unique DEGs. Gene ontology, Ingenuity Pathway Analysis (IPA), and enrichment analysis with Enrichr were performed to identify associated pathways, diseases and functions. Raw and processed data are available at Gene Expression Omnibus (GSE220060).

#### 4.8.17 RNA‐Seq

Library construction was performed using Takara SMARTer Stranded Total RNA‐Seq Kit—Pico Input Mammalian kit—V3, which is specifically designed for very low‐input total RNA samples. Clustering was done by ‘cBot,’ and samples were sequenced on NovaSeq6000 (NovaSeq Control Software 1.7.5/RTA v3.4.4) with a 151nt(Read1)‐19nt(Index1)‐10nt(Index2)‐151nt(Read2) setup using ‘NovaSeqXp’ workflow in ‘S4’ mode flowcell. The Bcl to FastQ conversion was performed using bcl2fastq_v2.20.0.422 from the CASAVA software suite. The quality scale used is Sanger/phred33/Illumina 1.8+. Processing of FASTQ files was carried out by the SciLifeLab National Genomics Infrastructure at the Uppsala Multidisciplinary Centre for Advanced Computational Science, Sweden. The sequenced reads were quality controlled with the FastQC software and pre‐processed with Trim Galore. The processed reads were then aligned to the reference genome of Mus musculus (build GRCm38) with the STAR aligner. Read counts for genes were generated using the feature Counts library and normalised TPM values calculated with StringTie, and raw gene read counts were generated by Salmon. Technical documentation on the RNA‐seq pipeline can be accessed here: https://github.com/nf‐core/rnaseq. Raw and processed data are available for download at Gene Expression Omnibus (https://www.ncbi.nlm.nih.gov/geo/) accession number: GSE220060.

#### 4.8.18 Differential Expression Analysis of genes in heart tissue

The raw gene count data generated from the Salmon tool, including 60,669 transcripts from three tissue samples each from HTB‐, HUVEC‐, CPC‐, LNP‐*VEGF‐A* mRNA treated mice, were imported into R for bioinformatic analysis, and statistical testing for differential expression was carried out using the DESeq2 R‐package41. Filtering and normalisation of the raw counts were performed for EVs of each cell line separately within the DESeq() function in the DESeq2 package. The Wald test was used for the identification of differentially expressed genes (DEGs). *p* values were adjusted for multiple testing using the Benjamini–Hochberg method, and an adjusted‐*p*‐value (adjP) of ≤0.05 was considered statistically significant. A log2 fold change (log_2_FC) shrinkage was carried out using ashr shrinkage estimator42, to reduce the variability of the lowly expressed genes. A result table with log_2_FC, *p*‐values and adjusted *p*‐values was generated. Genes with adjP  ≤ 0.05 and absolute log_2_FC > 1 are considered significant and are used for downstream functional and pathway analysis.

#### 4.8.19 Explorative Data Analysis

The gene expression dataset was further analysed to investigate the transcriptional effect of the injection of three different vesicles with *VEGF‐A* mRNA into heart tissues. The genes with no counts were filtered, and the data were normalised for each cell line using the varianceStabilizingTransformation() function in the DESeq2 package.

Principal component analysis (PCA) plots were generated from the top 500 most variable genes using plotPCA() function. The two axes, PC1 and PC2, represent the two principal components identified by the analysis. PC1 contributes 37% of the overall variation among samples and 21% for PC2. Additionally, the Volcano plots were generated using the EnhancedVolcano R package. Red and blue dots denote the significantly up‐ and down‐regulated genes passing adjusted *p* value and fold difference thresholds (–log10 of adjP‐value ≥ 1.3, abs(logFC)>1).

MA plots were generated using plotMA() function with a shrinkage estimator from ashr package42. The output from the logFC shrinkage was used for visualisation in MA plots. The top 1000 most variable genes were selected, and samples were clustered and visualised using heatmaps to assess the reproducibility and quality of the experiment. The pheatmap R‐package was used to create heatmaps with Spearman rank correlation as a distance measure.

#### 4.8.20 Venn Diagram for Overlapped and Unique DEGs

The DEGs between the untreated control mice and the mice injected with different EVs or LNPs were compared using VennDiagram R‐package to investigate the overlapped and unique DEGs in the heart tissue43. Functional analysis was carried out on 33 unique DEGs from the CPC treatment sample using Enrichr.

### 4.9 DEGs Associated With Inflammatory Response and Angiogenesis—Gene Ontology

A total of 364 and 266 unique Ensembl gene IDs associated with inflammatory response (GO:006954) and angiogenesis (GO:0001525) were identified using the biomaRt R package, respectively. The gene IDs associated with the two GO terms were compared to the IDs of DEGs to identify how many DEGs are associated with inflammatory response and angiogenesis.

### 4.10 Ingenuity Pathway Analysis

Differentially expressed genes (DEGs) with *p* ≤ 0.05 and |log_2_FC| > 1 were analysed using Ingenuity Pathway Analysis (IPA; Qiagen) 43. Core Analysis and Comparison Analysis were performed to identify enriched canonical pathways, diseases and biological functions, and gene interaction networks associated with the DEGs.

Statistical significance of canonical pathways and associated functions was determined using a right‐tailed Fisher's exact test (*p* ≤ 0.05). IPA also computes activation *Z*‐scores to predict pathway activity, where positive *Z*‐scores indicate activation and negative *Z*‐scores indicate inhibition. An absolute *Z*‐score ≥ 2 was considered indicative of significant pathway activation or inhibition.

### 4.11 Enrichment Analysis With Enrichr

Gene symbols of the differentially expressed genes (DEGs) were analysed using Enrichr44‐46. The top 10 enriched Gene Ontology (GO) Biological Processes (2021 release) and the top 10 MGI Mammalian Phenotype Level 4 terms (2021 release), ranked by *p* value, were selected. Bar plots displaying the top enriched terms and phenotypes as –log10(*p*‐values) were generated and exported.

### 4.12 Statistical Analysis

Statistical analysis was performed using GraphPad Prism version 10. For comparisons among multiple groups, statistical differences were evaluated using one‐way analysis of variance (ANOVA) or the Kruskal–Wallis test, as appropriate, to evaluate differences between treated and untreated samples. Where applicable, multiple comparisons were made using appropriate post hoc tests (e.g., Dunnett's test for comparisons against control or Dunn's test for non‐parametric data). Given the small sample sizes (*n* = 3), non‐parametric tests were applied when data did not consistently meet normality assumptions. Normality was assessed using multiple tests, including Shapiro–Wilk, D'Agostino–Pearson, Anderson–Darling and Kolmogorov–Smirnov tests. The specific statistical tests used for each dataset are indicated in the corresponding figure legends. Additionally, for RNA‐Seq data, the statistical analysis for DEGs was carried out using the DESeq2 R‐package41. Filtering and normalisation of the raw counts were performed for each EV type (in tissue) together within the DESeq() function in the DESeq2 package. The Wald test was used for the identification of DEGs. *p* values were adjusted for multiple testing using the Benjamini–Hochberg method. The differentially expressed genes with false discovery rate (FDR) rate of ≤0.05 and absolute log2 FC > 1 were considered statistically significant. A result table with log_2_ fold changes, *p* values, and FDR‐adjusted *p* values was generated and used for creating graphs. Statistical significance of canonical pathways (IPA network) and associated functions was determined using a right‐tailed Fisher's exact test (*p* ≤ 0.05). An absolute *Z*‐score ≥ 2 was considered indicative of significant pathway activation or inhibition.

## Author Contributions

Conceptualization: Hadi Valadi. Methodology: Muhammad Nawaz, Sepideh Heydarkhan‐Hagvall, Benyapa Tangruksa, John Wiseman, Franziska Kohl, Hernán González‐King Garibotti, Yujia Jing, Zahra Payandeh, Karin Jennbacken, Leif Hultin, Lennart Lindfors and Hadi Valadi. Investigation: Muhammad Nawaz, Sepideh Heydarkhan‐Hagvall, Lennart Lindfors, Jane Synnergren and Hadi Valadi. Data analysis: Muhammad Nawaz, Benyapa Tangruksa, Leif Hultin, Lennart Lindfors, Jane Synnergren and Hadi Valadi. Validation: Hadi Valadi and Muhammad Nawaz. Visualization: Hadi Valadi and Muhammad Nawaz. Funding acquisition: Hadi Valadi. Project administration: Hadi Valadi. Supervision: Hadi Valadi. Writing – original draft: Hadi Valadi and Muhammad Nawaz wrote the first draft with the input from all the co‐authors. Writing – review & editing: With the input from all the co‐authors.

## Conflicts of Interest

The authors declare no conflicts of interest. S.H.‐H., H.G.‐K., Y.J., K.J., J.W., L.H. and L.L. are all employed by AstraZeneca.

## Supporting information




**Supporting Information**: jev270324‐sup‐0001‐SuppMat.docx

**Supporting Information**:jev270324‐sup‐0002‐TableS1.xlsx

**Supporting Information**:jev270324‐sup‐0003‐TableS2.xlsx

**Supporting Information**:jev270324‐sup‐0004‐TableS3.xlsx

**Supporting Information**:jev270324‐sup‐0005‐TableS4.xlsx

**Supporting Information**:jev270324‐sup‐0006‐TableS5.xlsx

**Supporting Information**:jev270324‐sup‐0007‐TableS6.xlsx

**Supporting Information**:jev270324‐sup‐0008‐TableS7.xlsx

**Supporting Information**:jev270324‐sup‐0009‐TableS8.xlsx

**Supporting Information**:jev270324‐sup‐0010‐TableS9.xlsx

## Data Availability

All data associated with this study are presented in the main paper or the Supplementary Materials. The source data are available on request from the corresponding author. The RNA‐Seq data was deposited to the NCBI repository. The Raw and processed data of transcriptomics are available for download at Gene Expression Omnibus, NCBI (https://www.ncbi.nlm.nih.gov/geo/) accession number: GSE220060.
