# Supplementary material for: Targeted Delivery of mRNA to the Heart via Extracellular Vesicles or Lipid Nanoparticles
Source: J Extracell Vesicles. 2026 Jun 11;15(6):e70324. doi: 10.1002/jev2.70324 (PMC13257898; doi:10.1002/jev2.70324)
Supplement: Supplementary file 1 — Supporting Information: jev270324‐sup‐0001‐SuppMat.docx [file JEV2-15-e70324-s005.docx]

**Supplementary Figure Legends**

**Targeted delivery of mRNA to the heart via extracellular vesicles or lipid nanoparticles**

Muhammad Nawaz ^1^, Benyapa Tangruksa ^1,3^, Sepideh Heydarkhan-Hagvall ^2,3^, Franziska Kohl ^4,5^, Hernán González-King Garibotti ^6^, Yujia Jing ^7^, Zahra Payandeh ^1^, Azadeh Reyahi ^1^, Karin Jennbacken ^6^, John Wiseman ^4^, Leif Hultin ^8^, Lennart Lindfors ^7^, Jane Synnergren ^3,9^, Hadi Valadi ^1^*

**Affiliations**

^1^ Department of Rheumatology and Inflammation Research, Institute of Medicine, Sahlgrenska Academy, University of Gothenburg, Gothenburg, 41346, Sweden

^2^ Chief Medical Office, Global Patient Safety, BioPharmaceuticals R&D, AstraZeneca, Gaithersburg MD, USA

^3^ Systems Biology Research Center, School of Bioscience, University of Skövde, SE-541 28 Skövde, Sweden

^4^ Centre for Genomics Research, Discovery Sciences, BioPharmaceuticals R&D, AstraZeneca, Gothenburg 43183 Mölndal, Sweden

^5^ Department of Medical Biochemistry and Biophysics, Karolinska Institute, Solna, Stockholm, 171 77, Sweden

^6^ Bioscience Cardiovascular, Research and Early Development, Cardiovascular, Renal and Metabolism (CVRM), BioPharmaceuticals R&D, AstraZeneca, 431 83, Gothenburg, Mölndal, Sweden

^7^ Advanced Drug Delivery, Pharmaceutical Sciences, BioPharmaceuticals R&D, AstraZeneca, Gothenburg, 431 83 Mölndal, Sweden

^8^ Discovery Imaging, Clinical Pharmacology and Safety Sciences, BioPharmaceuticals R&D, AstraZeneca, Gothenburg, 431 83 Mölndal, Sweden

^9^ Department of Molecular and Clinical Medicine, Institute of Medicine, Sahlgrenska Academy, University of Gothenburg, Gothenburg, 41345, Sweden

^*^Correspondence: Hadi Valadi ([hadi.valadi@gu.se](mailto:hadi.valadi@gu.se))

**
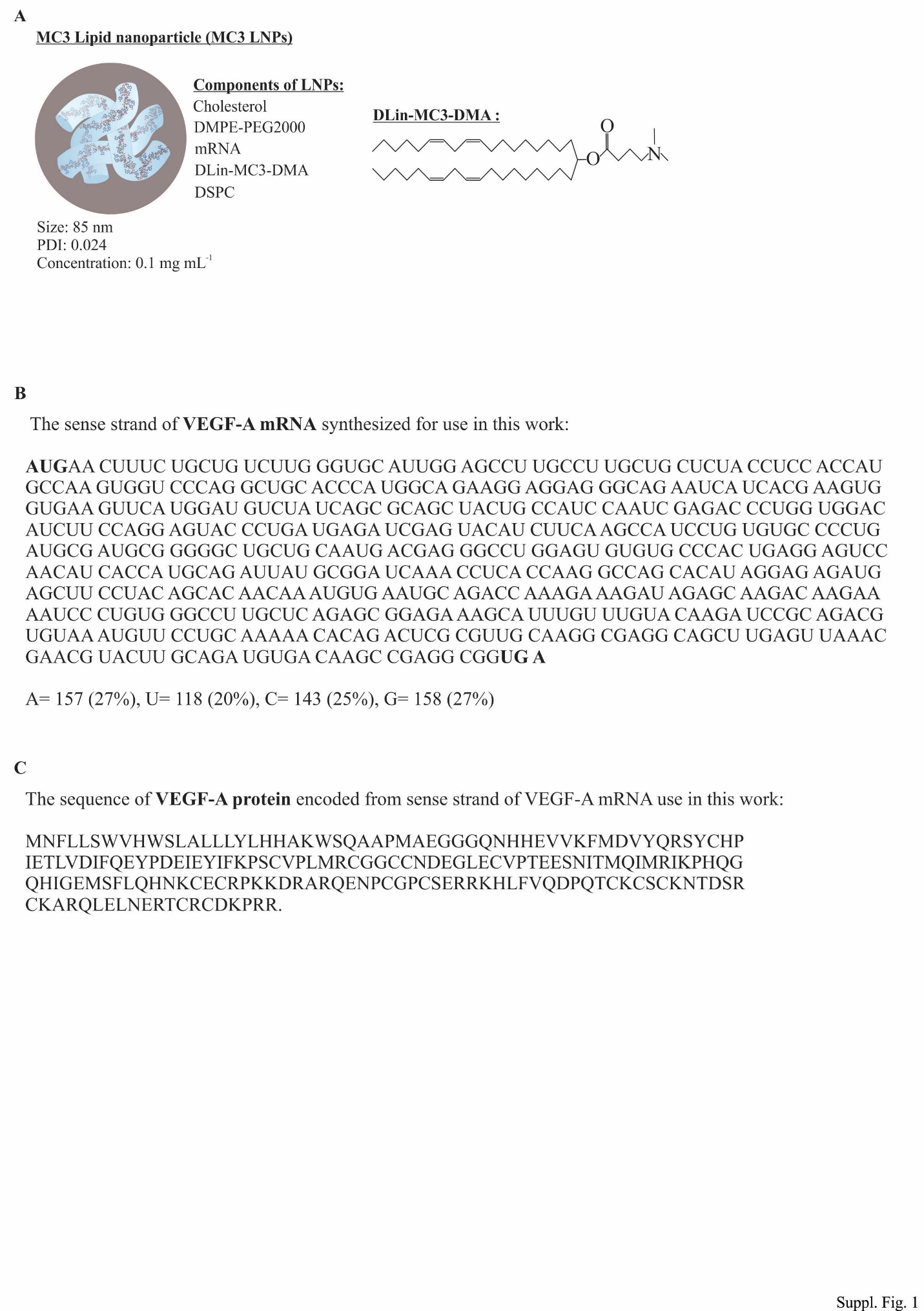
**

**Supplementary Figure 1. DLin-MC3-DMA lipid nanoparticles (MC3-LNPs) and *VEGF-A* mRNA and protein sequences used in the current study.** **(A)** Chemical structure and components of DLin-MC3-DMA LNPs (MC3-LNPs) including encapsulated mRNA. **(B)** Sense strand sequence of the synthesized CleanCap *VEGF-A* mRNA (isoform 11). **(C)** Amino acid sequence of VEGF-A protein encoded from the *VEGF-A* mRNA. PDI: polydispersity index.


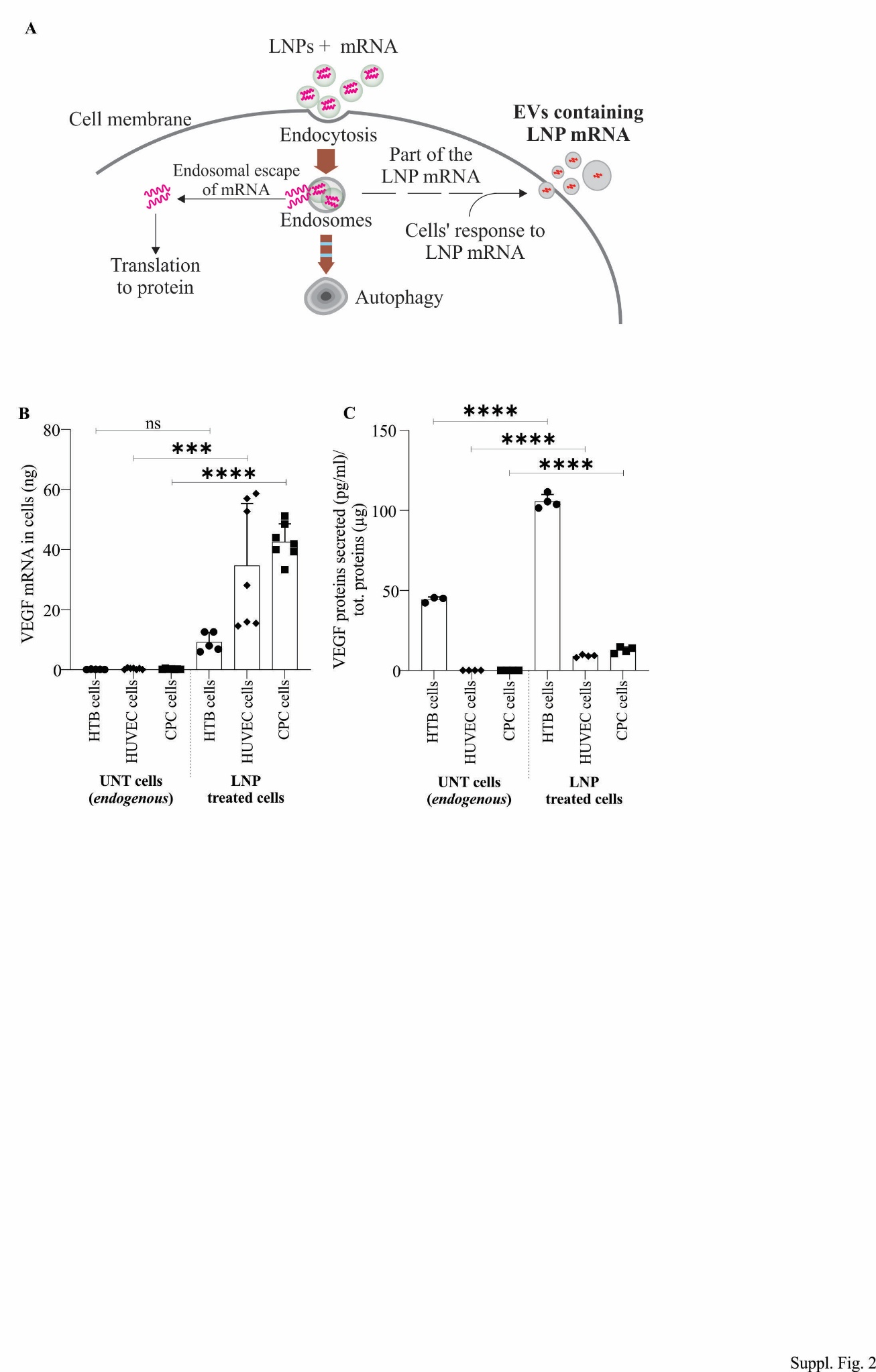


**Supplementary Figure 2.** **Cytoplasmic delivery** **of *VEGF-A* mRNA via LNPs**

**(A)** Schematic illustration of the cytoplasmic uptake of LNP-mRNA and translation into protein or incorporation into EVs after endosomal escape. A total of 3 µg of LNP-*VEGF-A* mRNA was administered to cardiac progenitor cells (CPCs), human umbilical vein endothelial cells (HUVECs), and a human lung epithelial cell line (HTB cells). (B) qPCR-based detection and quantification of *VEGF-A* mRNA in the lysates of recipient CPCs, HUVECs, and HTB cells. (C) Quantification of VEGF-A protein in the supernatants of corresponding cells by ELISA. Statistical comparisons among groups were performed using one-way ANOVA. Statistical significance is indicated as ****p < 0.0001; ns, not significant. Data are presented as mean ± SD of n = 5, 7, and 7 (untreated HTB, HUVEC, and CPC, respectively) and n = 5, 7, and 7 (treated HTB, HUVEC, and CPC, respectively) for *VEGF-A* mRNA. For VEGF-A protein, n = 3, 4, and 4 (untreated HTB, HUVEC, and CPC, respectively) and n = 4 for treated groups.

**
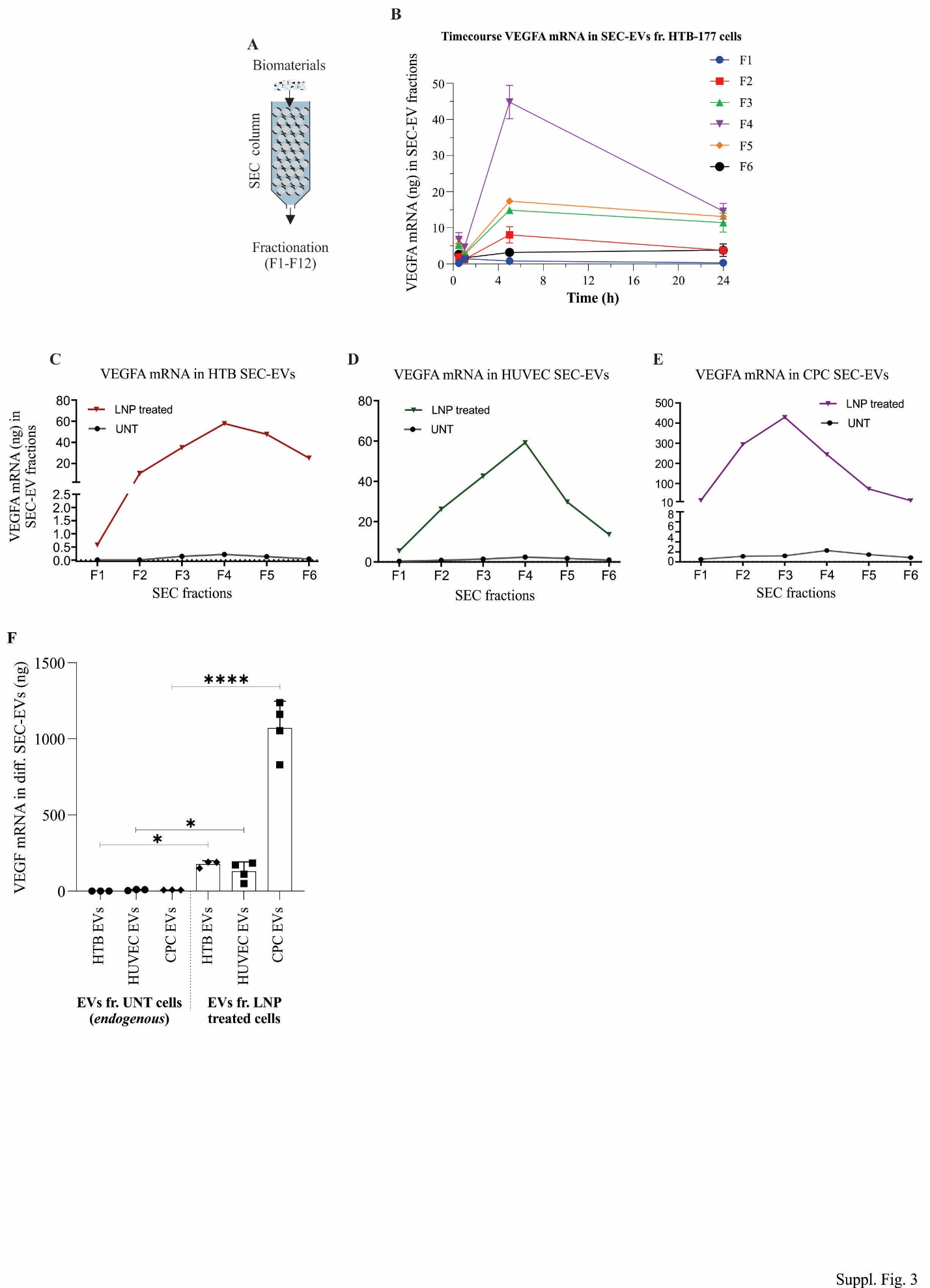
**

**Supplementary Figure 3.** **Detection and quantification of *VEGF-A* mRNA in SEC-isolated EVs from three cell types over time**

**(A)** Schematic illustrating the isolation of EVs by size exclusion chromatography (SEC). A total of 15 mL of conditioned media collected at indicated time points after treatment was loaded onto a qEV/10 SEC column. Following passage of the void volume, twelve SEC fractions were collected. **(B)** Time course of *VEGF-A* mRNA levels quantified by qPCR in HTB-derived SEC-EVs. **(C–E)** *VEGF-A* mRNA levels quantified in SEC-EVs from cardiac progenitor cells (CPC-EVs), human umbilical vein endothelial cells (HUVEC-EVs), and HTB cells (HTB-EVs). SEC fractions from untreated cells were used as controls, showing no or negligible *VEGF-A* mRNA levels. **(F)** Total amounts of *VEGF-A* mRNA in the pooled SEC-EV fractions (F1–F6). Statistical comparisons among groups were performed using one-way ANOVA. Statistical significance is indicated as *p < 0.05 and ****p < 0.0001; ns, nonsignificant. Data are presented as mean ± SD of n = 4 biological replicates, except for untreated groups where n = 3.

**
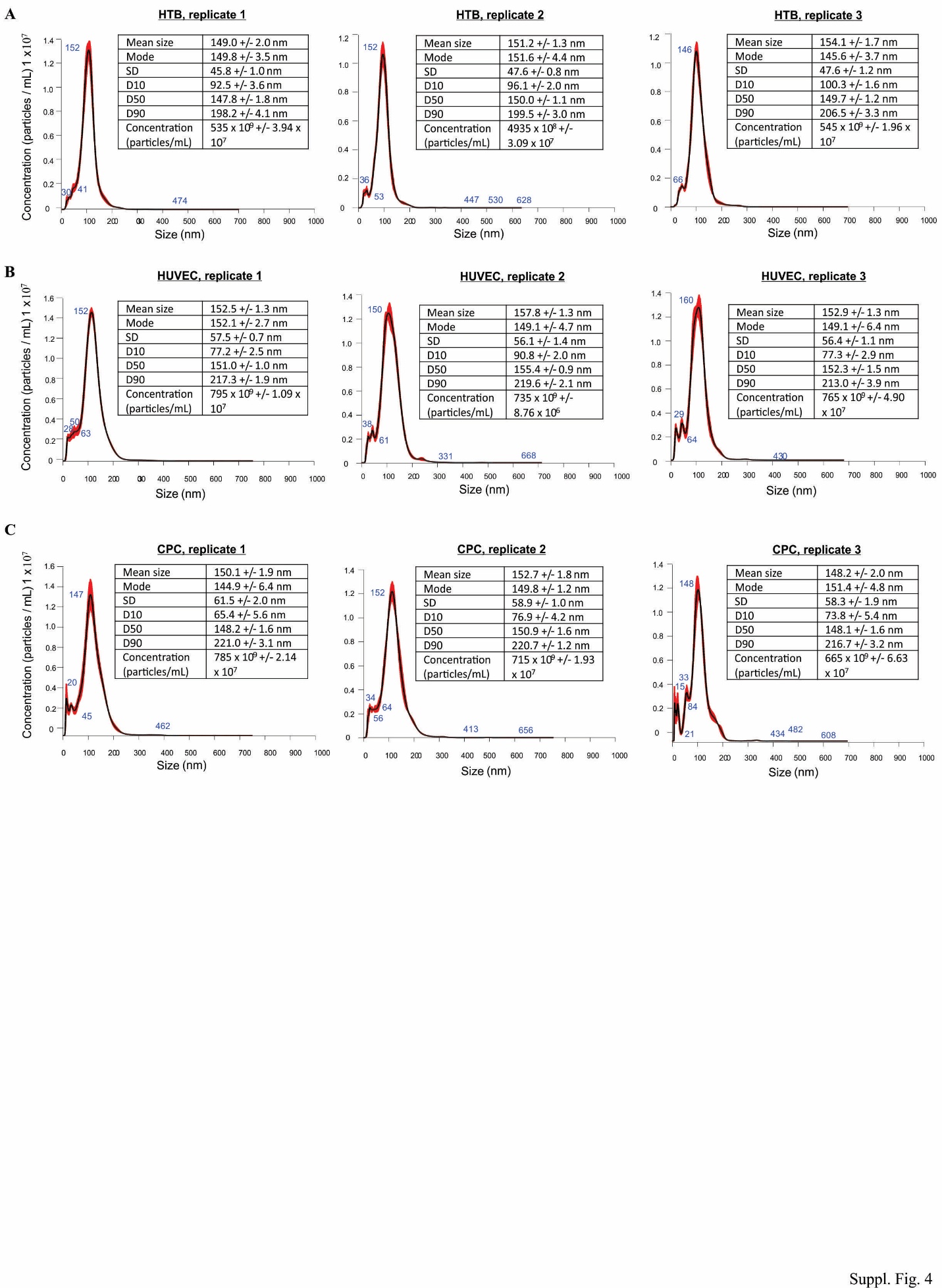
**

**Supplementary Figure 4.** **Size distribution and particle concentration of EVs.** Nanoparticle tracking analysis (NTA)-based size distribution and particle concentration of **(A)** HTB SEC-EVs, **(B)** HUVEC SEC-EVs, and **(C)** CPC SEC-EVs. Samples were diluted 10-fold. The graphs were obtained from diluted samples, and the values were corrected by the dilution factor (×10). The adjusted values are presented in tables associated with each graph.

**
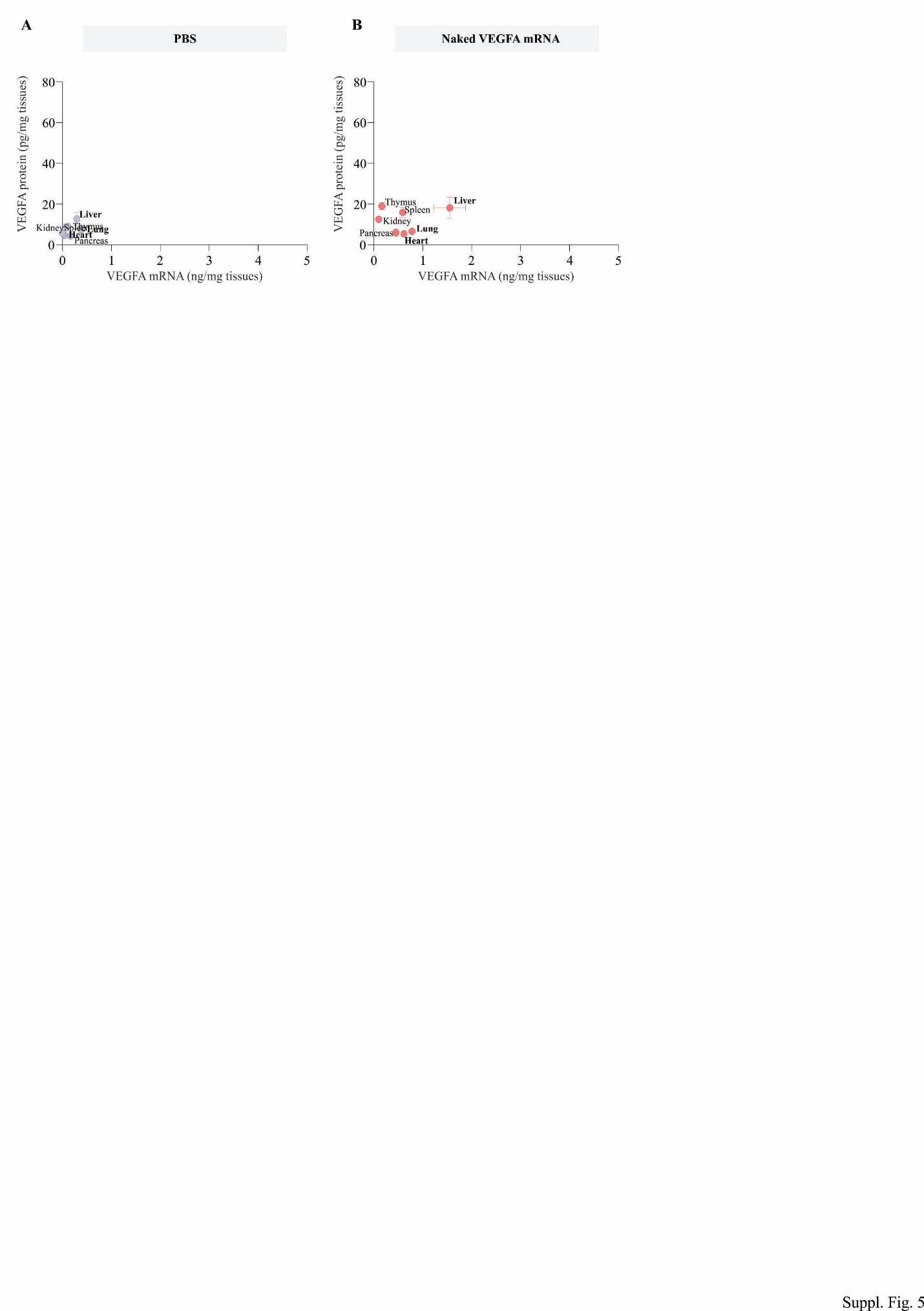
**

**Supplementary Figure 5.** **Organ-specific localization of *VEGF-A mRNA* and protein following naked mRNA delivery**

Quantitative analysis of organ-specific distribution demonstrating the relationship between *VEGF-A* mRNA levels and corresponding VEGF-A protein levels across organs following intravenous administration of **(A)** PBS or **(B)** naked *VEGF-A* mRNA. *VEGF-A* mRNA levels are expressed as ng/mg tissue, and VEGF-A protein levels are expressed as pg/mg tissue.


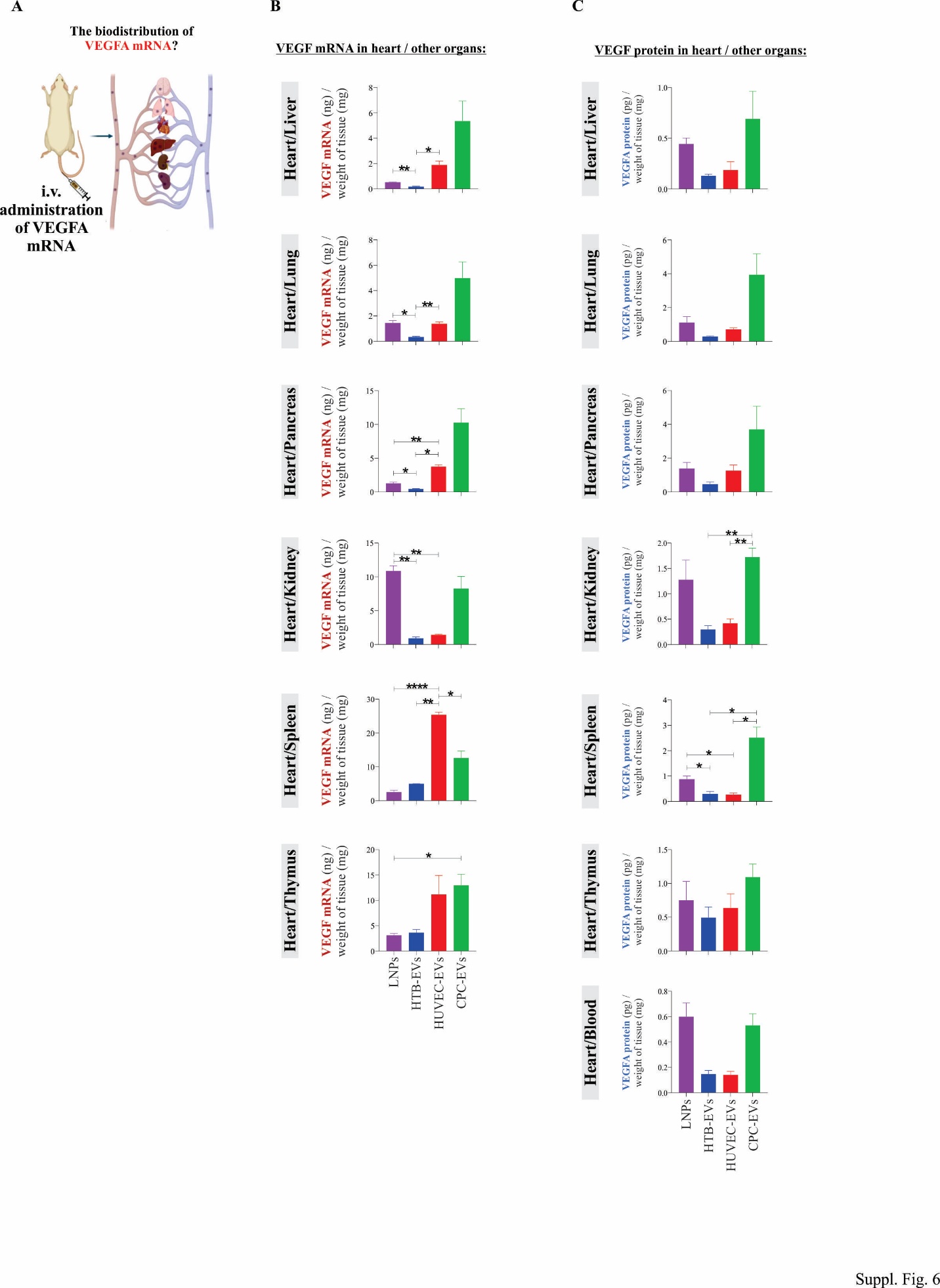


**Supplementary Figure 6.** **Heart-to-organ ratios of *VEGF-A* mRNA and protein levels following intravenous delivery via EVs or LNPs**

**(A)** 1 µg of *VEGF-A* mRNA was delivered to C57BL/6Ncrl mice intravenously via CPC-EVs, HTB-EVs, HUVEC-EVs, or LNPs. Mice were euthanized 6 h after administration, and organs were collected to assess *VEGF-A* mRNA biodistribution. **(B)** Heart-to-organ ratios of *VEGF-A* mRNA levels in the heart relative to other organs (heart/organ). **(C)** Heart-to-organ ratios of VEGF-A protein levels in the heart relative to other organs and blood (heart/organ). Statistical comparisons among groups were performed using one-way ANOVA. Statistical significance is indicated as *p < 0.05, **p < 0.01, ****p < 0.0001. Only statistically significant differences (p ≤ 0.05) are displayed. *VEGF-A* mRNA levels are expressed as ng/mg tissue. Data are presented as mean ± SD of n = 3 biological replicates per group. HTB-EVs: HTB-177 lung epithelial cell-derived EVs; HUVEC-EVs: human umbilical vein endothelial cell-derived EVs; CPC-EVs: cardiac progenitor cell-derived EVs.


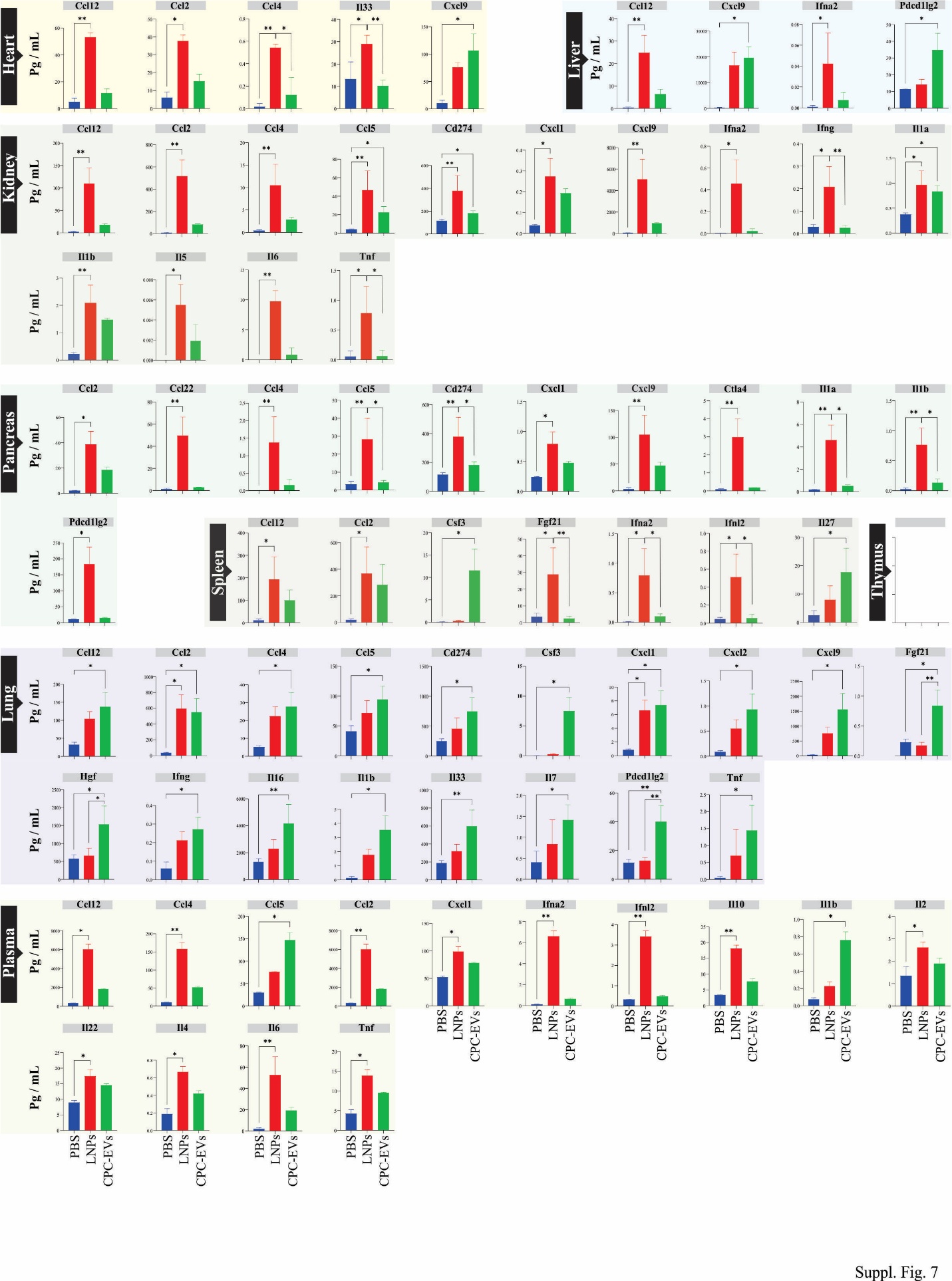


**Supplementary Figure 7. Organ-specific cytokine responses induced by LNPs and CPC-EVs.** Tissue and plasma samples were collected 6 h after intravenous administration of *VEGF-A* mRNA via lipid nanoparticles (LNPs) or CPC-derived extracellular vesicles (CPC-EVs). Cytokine levels were quantified using the Olink® Target 48 Mouse Cytokine multiplex panel, based on proximity extension assay (PEA) technology. Samples were processed and analyzed by Olink Proteomics.
Protein abundance was reported as normalized protein expression (NPX; log2 scale, relative quantification) and, where available, as absolute concentrations (pg/mL).

Internal and external controls provided by Olink were used for quality control and normalization according to the manufacturer’s standard data processing pipeline. Cytokine profiles across organs and plasma following LNP- or CPC-EV-mediated delivery are shown. LNP-mediated delivery resulted in significant upregulation of multiple cytokines across several organs, including liver, plasma, lung, kidney, and heart, whereas CPC-EV-mediated delivery induced fewer cytokine changes that were predominantly observed in the lung, with fewer changes in other organs. Direct comparisons between LNPs and CPC-EVs are shown for cytokines exhibiting significant differences between the delivery platforms. Statistical comparisons among groups (LNPs or CPC-EVs with PBS, as well as between LNPs or CPC-EVs) were performed using the Kruskal-Wallis test. Statistical significance is indicated as *p < 0.05 and **p < 0.01. Only statistically significant differences (p ≤ 0.05) are displayed. Data are presented as mean ± SD of n = 3 biological replicates per group. LNPs: lipid nanoparticles; CPC-EVs: cardiac progenitor cell-derived EVs.


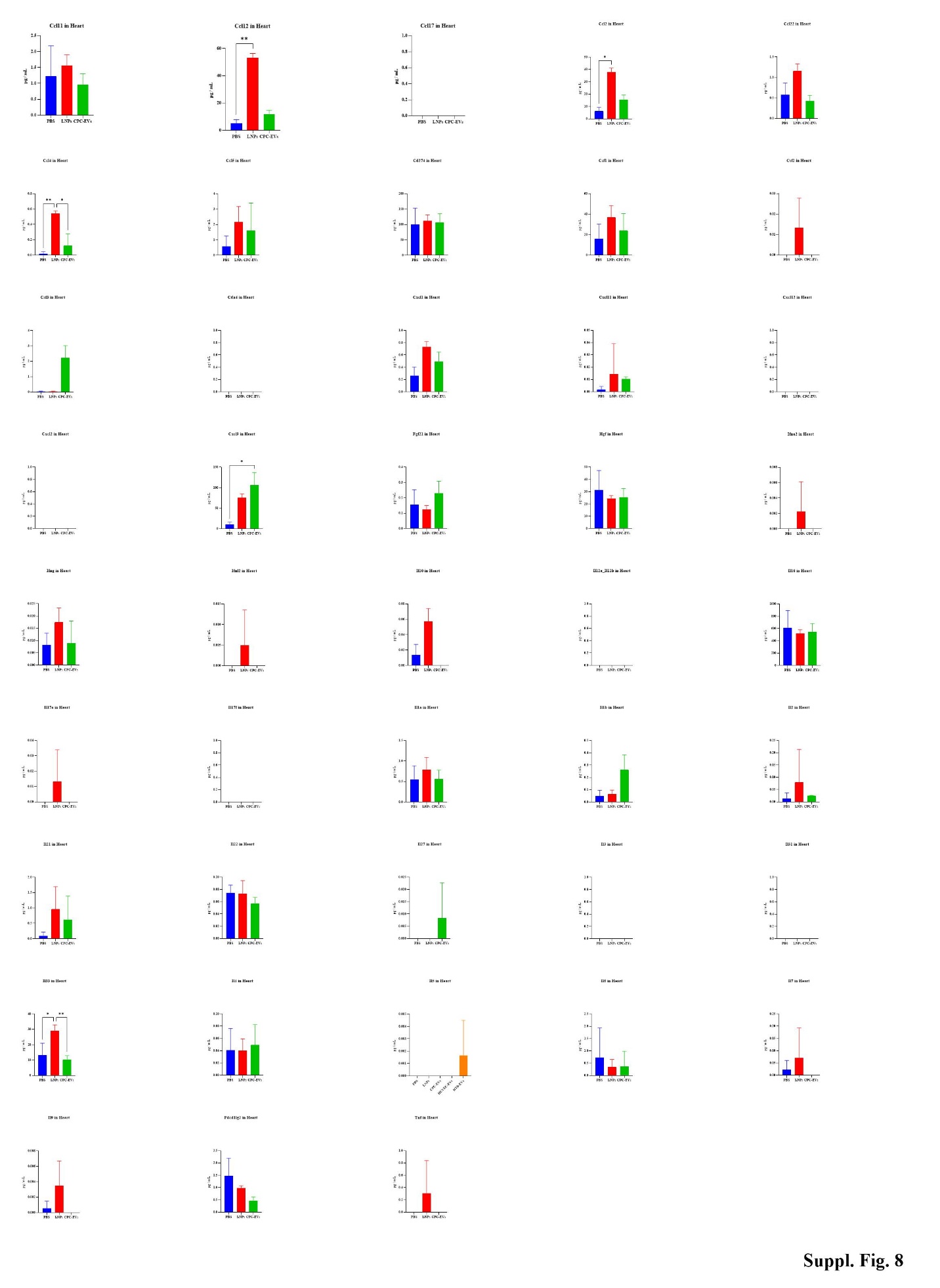


**Supplementary Figure 8.** **Complete expression profile of cytokines quantified in heart tissue after mRNA delivery via LNPs or CPC-EVs compared with PBS-treated controls.** Cytokine levels were quantified using the Olink® Target 48 Mouse Cytokine panel based on proximity extension assay (PEA) technology. Protein abundance was reported as normalized protein expression (NPX; log₂ scale) and, where available, as absolute concentrations (pg/mL). Internal and external controls provided by Olink were used for quality control and normalization according to the manufacturer’s standard pipeline. Statistical comparisons among groups were performed using one-way ANOVA. Statistical significance is indicated as *p < 0.05, ***p < 0.001, and ****p < 0.0001. Only statistically significant differences (p ≤ 0.05) are displayed. Data are presented as mean ± SD of n = 3 biological replicates per group.


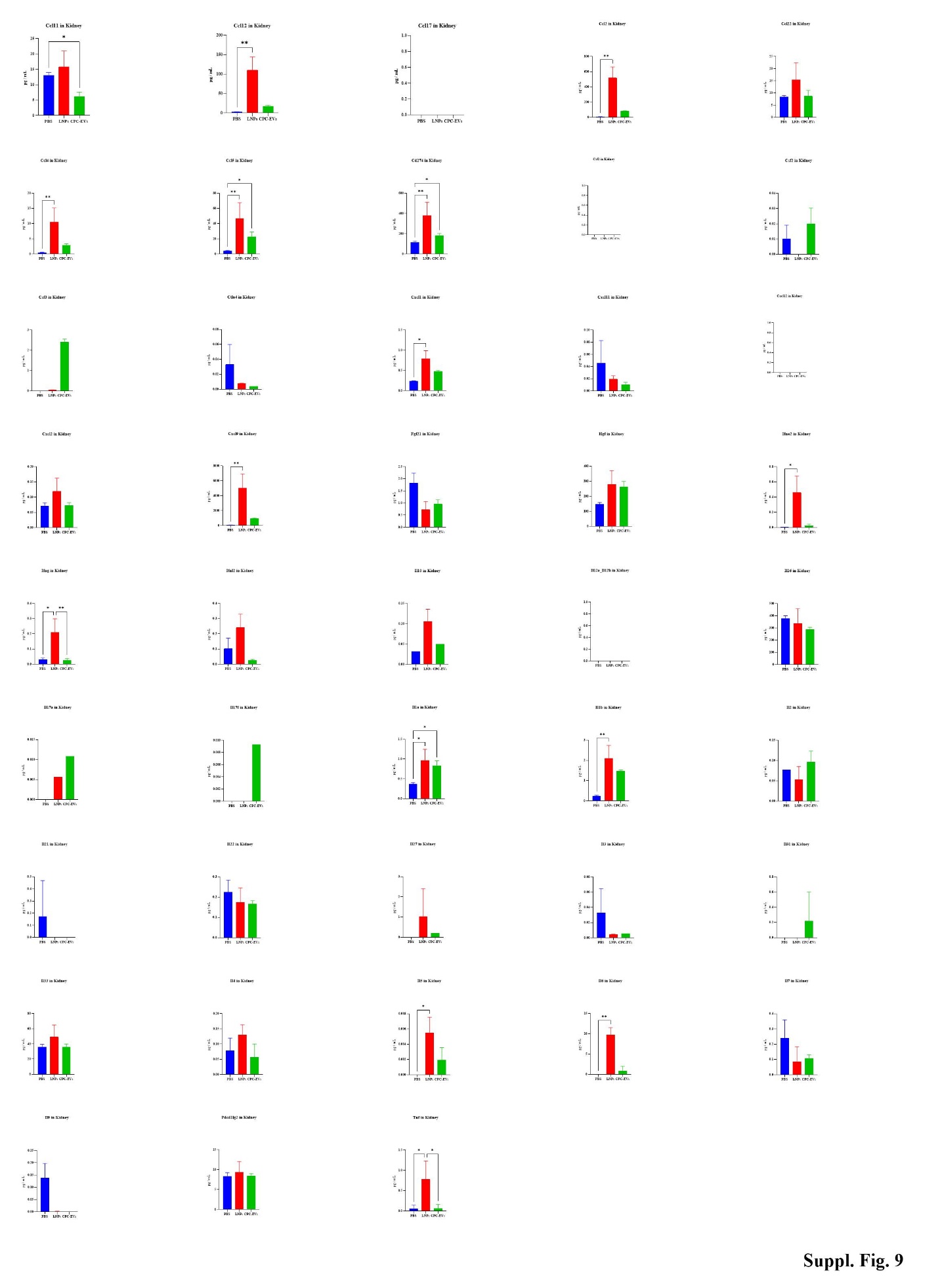


**Supplementary Figure 9. Complete expression profile of cytokines quantified in the kidney after mRNA delivery via LNPs or CPC-EVs compared with PBS-treated controls.** Statistical comparisons among groups were performed using one-way ANOVA. Statistical significance is indicated as *p < 0.05, **p < 0.01, ***p < 0.001, and ****p < 0.0001. Only statistically significant differences (p ≤ 0.05) are displayed. Data are presented as mean ± SD (n = 3 biological replicates per group).


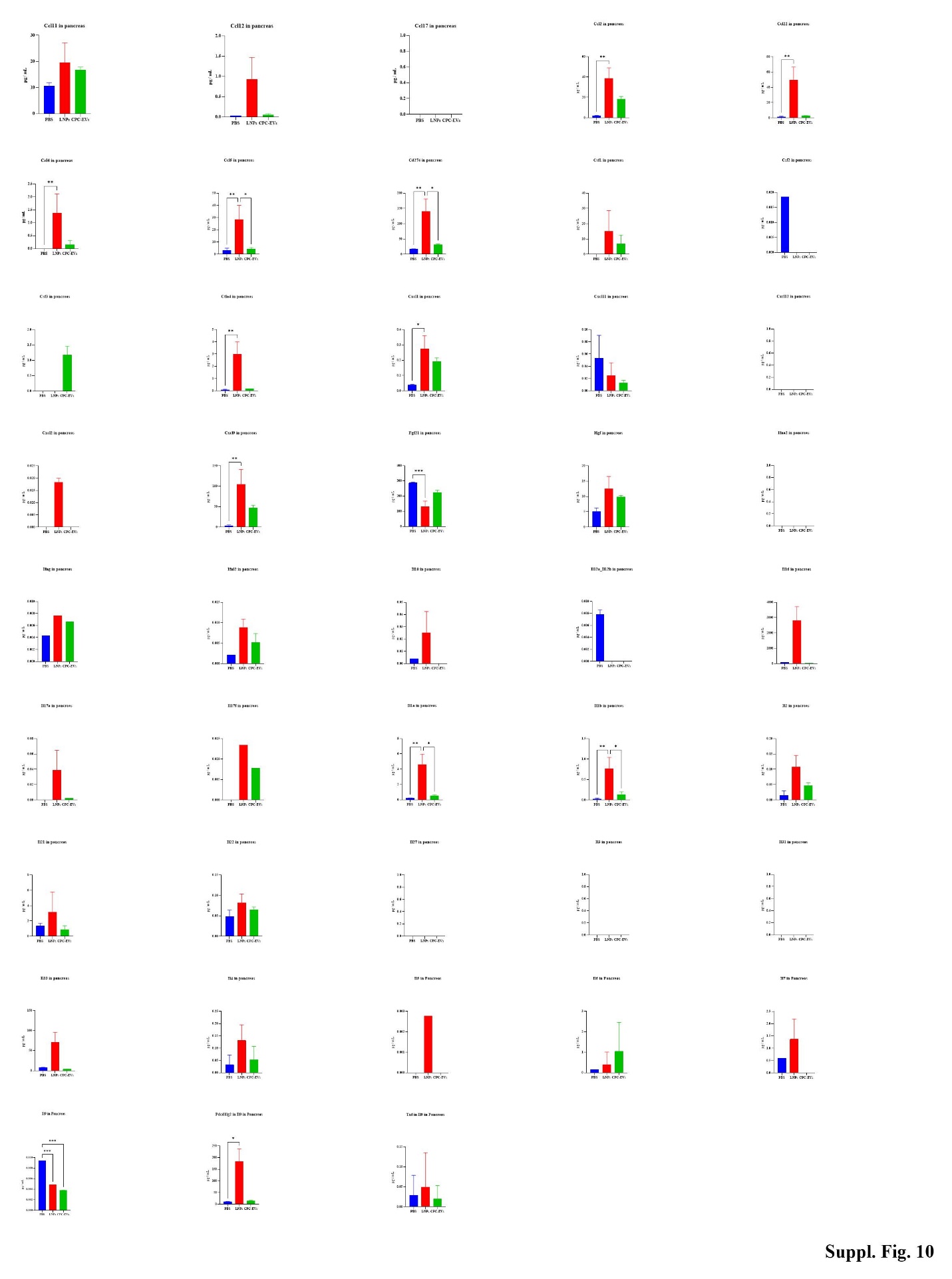


**Supplementary Figure 10.** **Complete expression profile of cytokines quantified in the pancreas after mRNA delivery via LNPs or CPC-EVs compared with PBS-treated controls.** Statistical significance is indicated as *p < 0.05, **p < 0.01, ***p < 0.001, and ****p < 0.0001. Only statistically significant differences (p ≤ 0.05) are displayed. Data are presented as mean ± SD (n = 3 biological replicates per group).


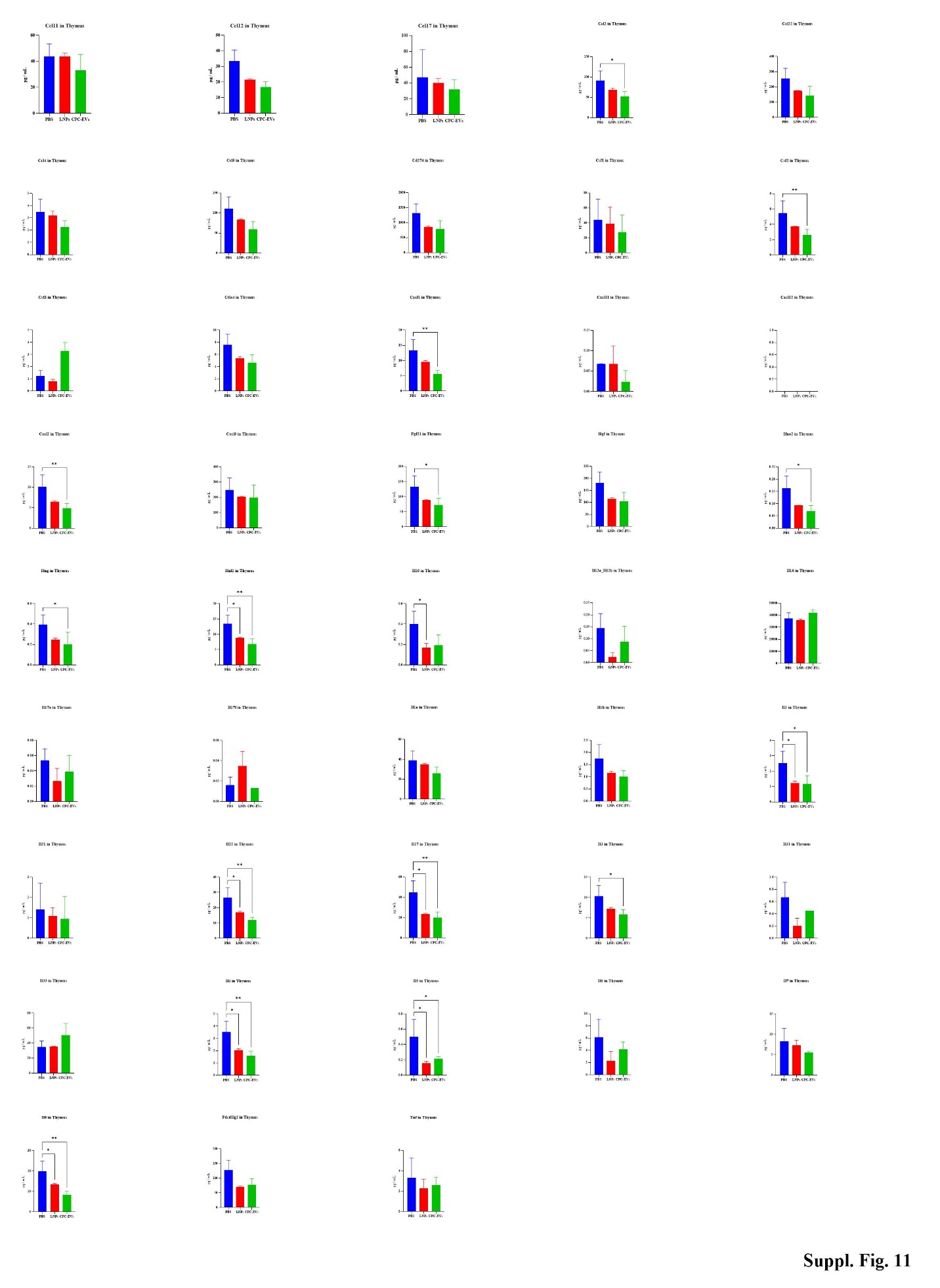


**Supplementary Figure 11.** **Complete expression profile of cytokines quantified in the thymus after mRNA delivery via LNPs or CPC-EVs compared with PBS-treated controls.** Statistical significance is indicated as *p < 0.05, and **p < 0.01. Only statistically significant differences (p ≤ 0.05) are displayed. Data are presented as mean ± SD (n = 3 biological replicates per group).


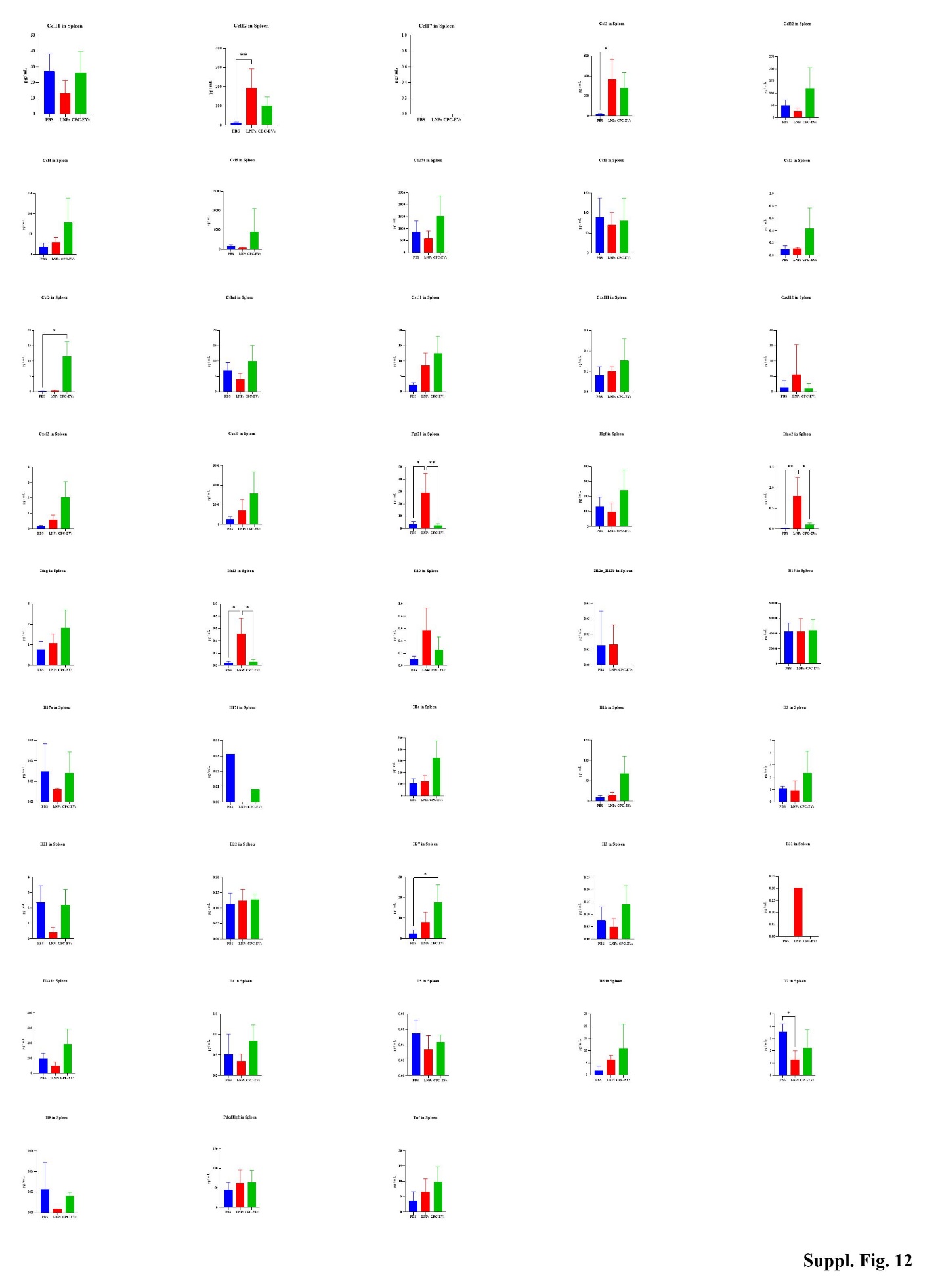


**Supplementary Figure 12.** **Complete expression profile of cytokines quantified in the spleen after mRNA delivery via LNPs or CPC-EVs compared with PBS-treated controls.** Statistical significance is indicated as *p < 0.05, and **p < 0.01. Only statistically significant differences (p ≤ 0.05) are displayed. Data are presented as mean ± SD (n = 3 biological replicates per group).


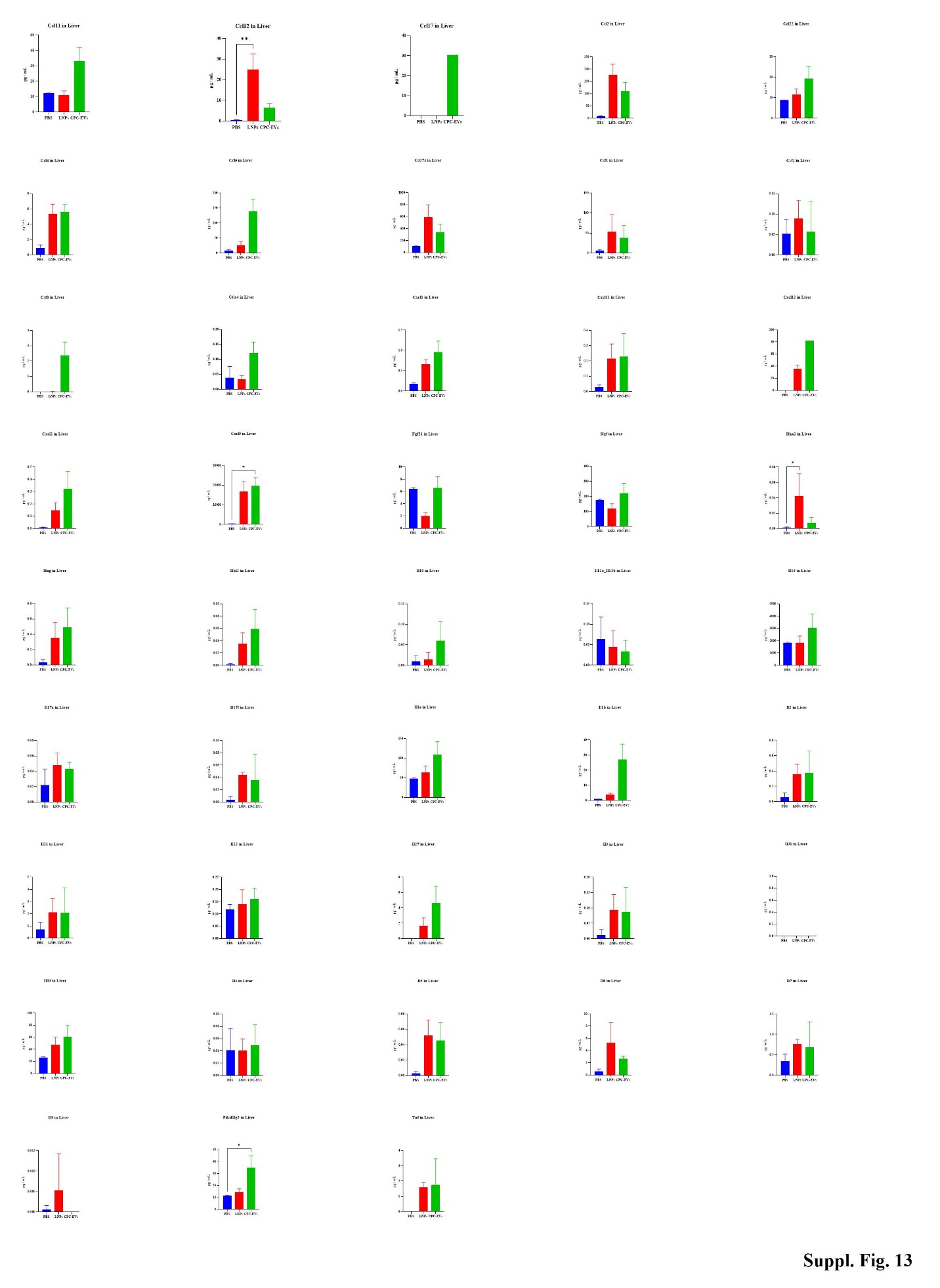


**Supplementary Figure 13.** **Complete expression profile of cytokines quantified in the liver after mRNA delivery via LNPs or CPC-EVs compared with PBS-treated controls.** Statistical significance is indicated as *p < 0.05, **p < 0.01, ***p < 0.001, and ****p < 0.0001. Only statistically significant differences (p ≤ 0.05) are displayed. Data are presented as mean ± SD (n = 3 biological replicates per group).


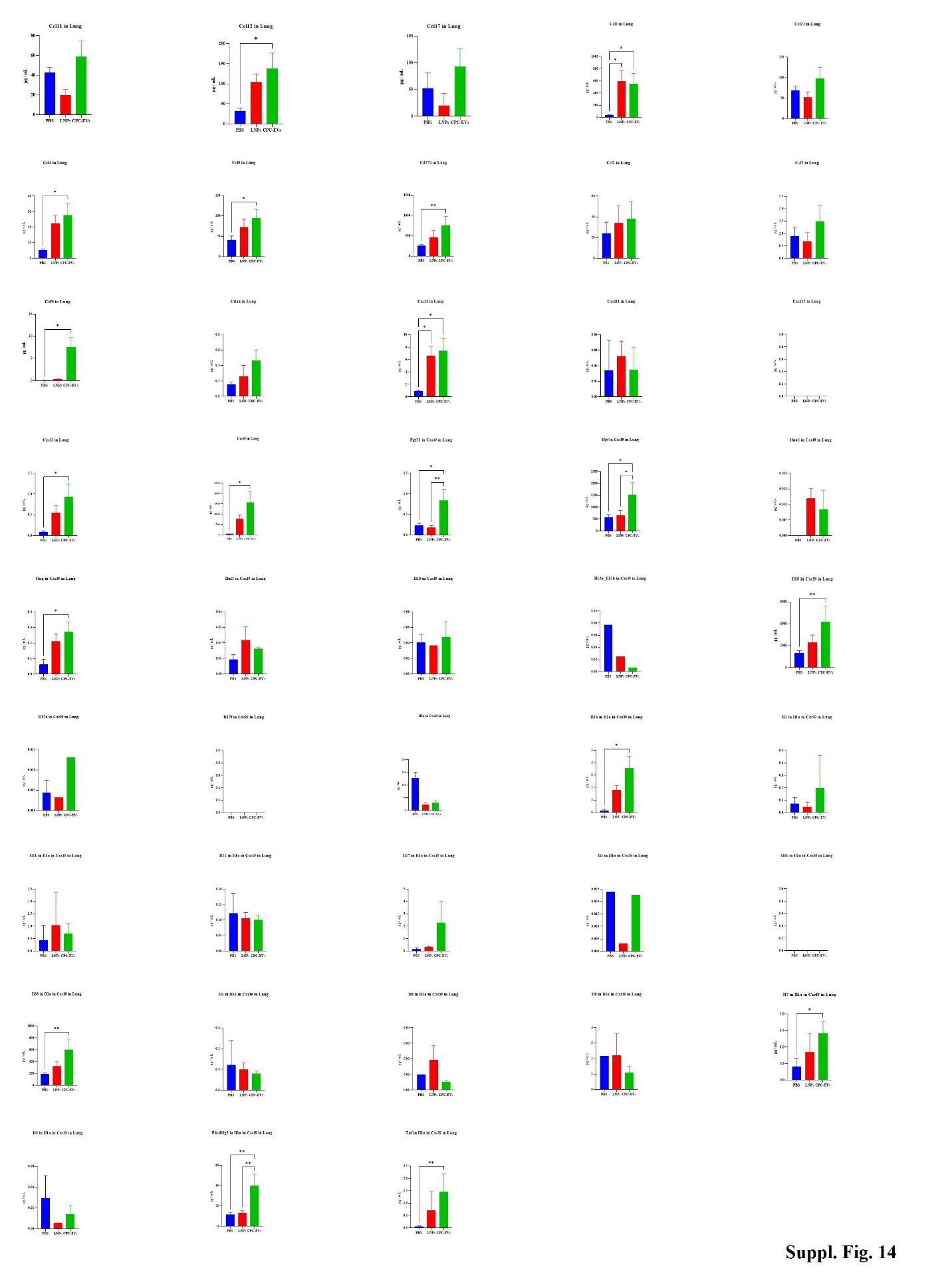


**Supplementary Figure 14.** **Complete expression profile of cytokines quantified in the lung after mRNA delivery via LNPs or CPC-EVs compared with PBS-treated controls.** Statistical significance is indicated as *p < 0.05, **p < 0.01, ***p < 0.001, and ****p < 0.0001. Only statistically significant differences (p ≤ 0.05) are displayed. Data are presented as mean ± SD (n = 3 biological replicates per group).


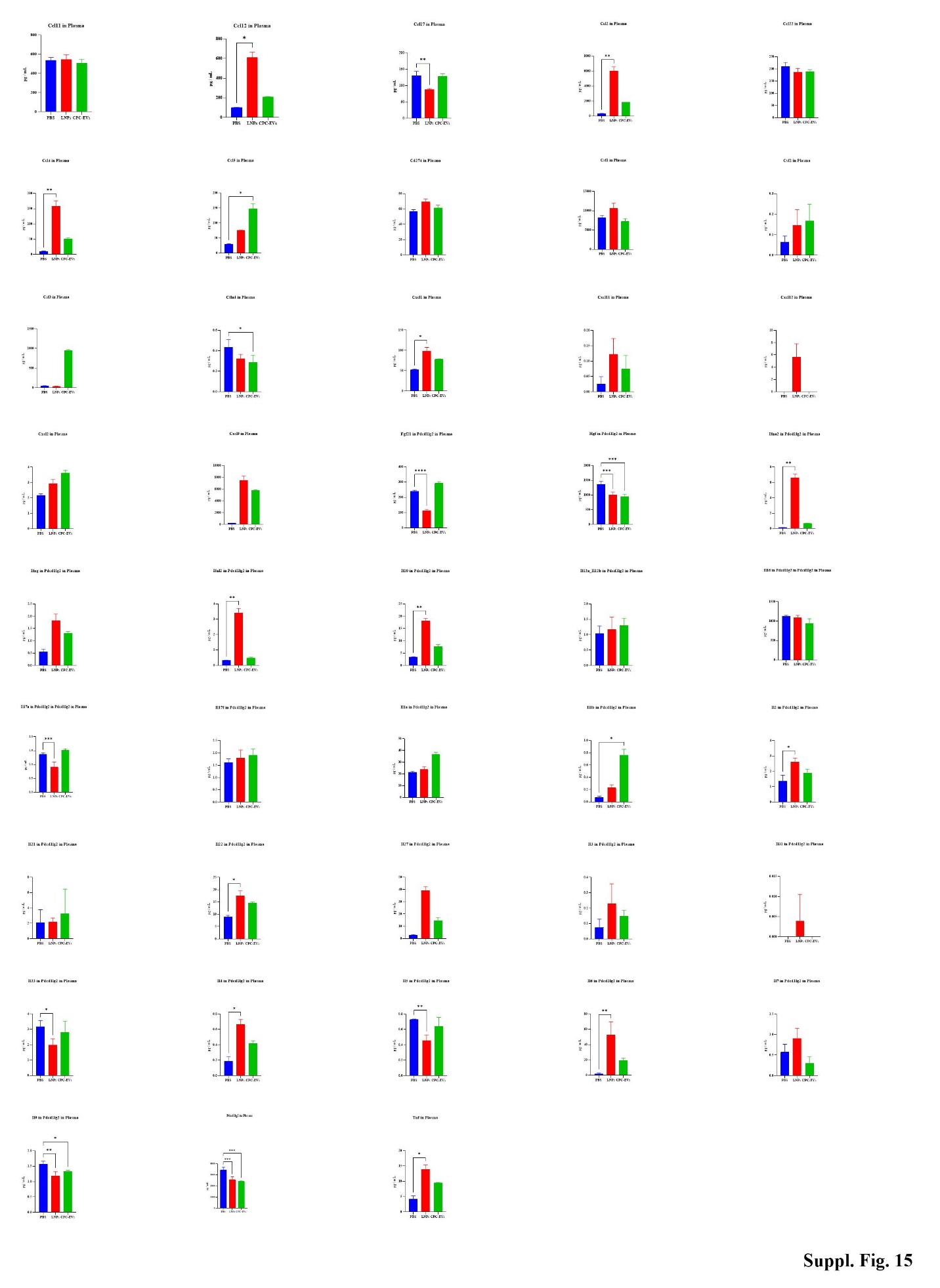


**Supplementary Figure 15.** **Complete expression profile of cytokines quantified in plasma after mRNA delivery via LNPs or CPC-EVs compared with PBS-treated controls.** Statistical significance is indicated as *p < 0.05, **p < 0.01, ***p < 0.001, and ****p < 0.0001. Only statistically significant differences (p ≤ 0.05) are displayed. Data are presented as mean ± SD (n = 3 biological replicates per group).


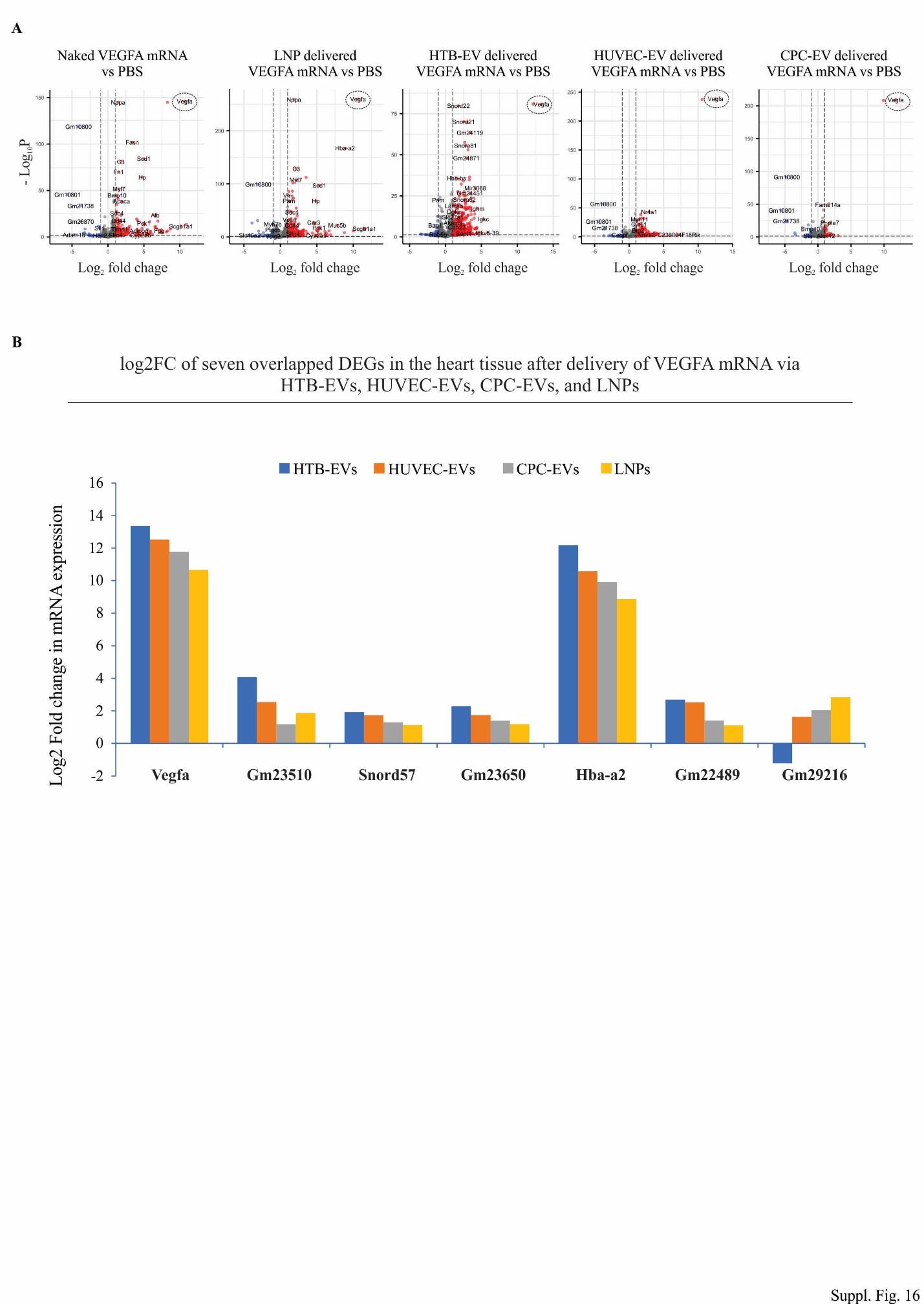


**Supplementary Figure 16. Differential expression and comparison of shared DEGs in cardiac tissue following *VEGF-A* mRNA delivery via EVs or LNPs.** **(A)** Volcano plots show dysregulated genes in the tissue after the injection, identified using transcriptomic data. Red and blue dots denote the significant up- and down-regulated genes passing adjusted P value and fold difference thresholds (-log_10_ of adjP-value ≥ 1.3, abs(logFC)>1). **(B)** Shared genes between CPC-EVs, HTB-EVs, HUVEC-EVs and LNPs after *VEGF-A* mRNA delivery to heart tissue. Log2 fold changes (log_2_FC) in mRNA expression are shown. CPC-EVs: cardiac progenitor cell-derived extracellular vesicles (EVs), HTB-EVs: HTB-177 lung epithelial cell-derived EVs, HUVEC-EVs: human umbilical vein endothelial cell-derived EVs, LNPs: lipid nanoparticles.

**Supplementary Figure 17. Ingenuity Pathway Analysis (IPA) of differentially expressed genes (DEGs) in cardiac tissue following intramyocardial delivery of *VEGF-A* mRNA via CPC-EVs**. CPC-EVs predominantly activate pathways linked to regenerative and cardiogenic processes with limited inflammatory signaling. Node colors indicate relative gene expression changes (red: upregulated; green: downregulated), and edge types represent predicted molecular interactions.

**Supplementary Figure 18.** **Ingenuity Pathway Analysis (IPA) of differentially expressed genes (DEGs) in cardiac tissue following intramyocardial delivery of *VEGF-A* mRNA via HTB-EVs.** The top three networks are shown based on network scores. These networks are associated with metabolic and translational processes, cardiovascular disease and cell death/survival, and inflammatory and immunological responses. Compared to CPC-EVs, HTB-EVs induce broader and more inflammatory transcriptional activation. Node colors represent gene expression changes (red: upregulated; green: downregulated), and connecting lines indicate predicted functional interactions.

**Supplementary Figure 19. Ingenuity Pathway Analysis (IPA) of differentially expressed genes (DEGs) in cardiac tissue following intramyocardial delivery of *VEGF-A* mRNA via HUVEC-EVs.** The top three networks are presented, highlighting pathways related to metabolic and mitochondrial processes, cellular development and proliferation, and vascular and smooth muscle function. These networks reflect an intermediate transcriptional profile, with features of both regenerative signaling and immune/inflammatory activation. Node colors indicate relative expression changes (red: upregulated; green: downregulated), and edges represent predicted molecular relationships.

**Supplementary Figure 20. Ingenuity Pathway Analysis (IPA) of differentially expressed genes (DEGs) in cardiac tissue following intramyocardial delivery of *VEGF-A* mRNA via LNPs.** The top three networks are shown, ranked by network score. These networks are predominantly associated with cell-to-cell signaling and interaction, cellular growth and proliferation, inflammatory responses, and immune cell trafficking. Compared to EV-mediated delivery, LNPs induce broader activation of inflammatory and stress-related pathways. Node colors indicate gene expression changes (red: upregulated; green: downregulated), and edges represent predicted molecular interactions.


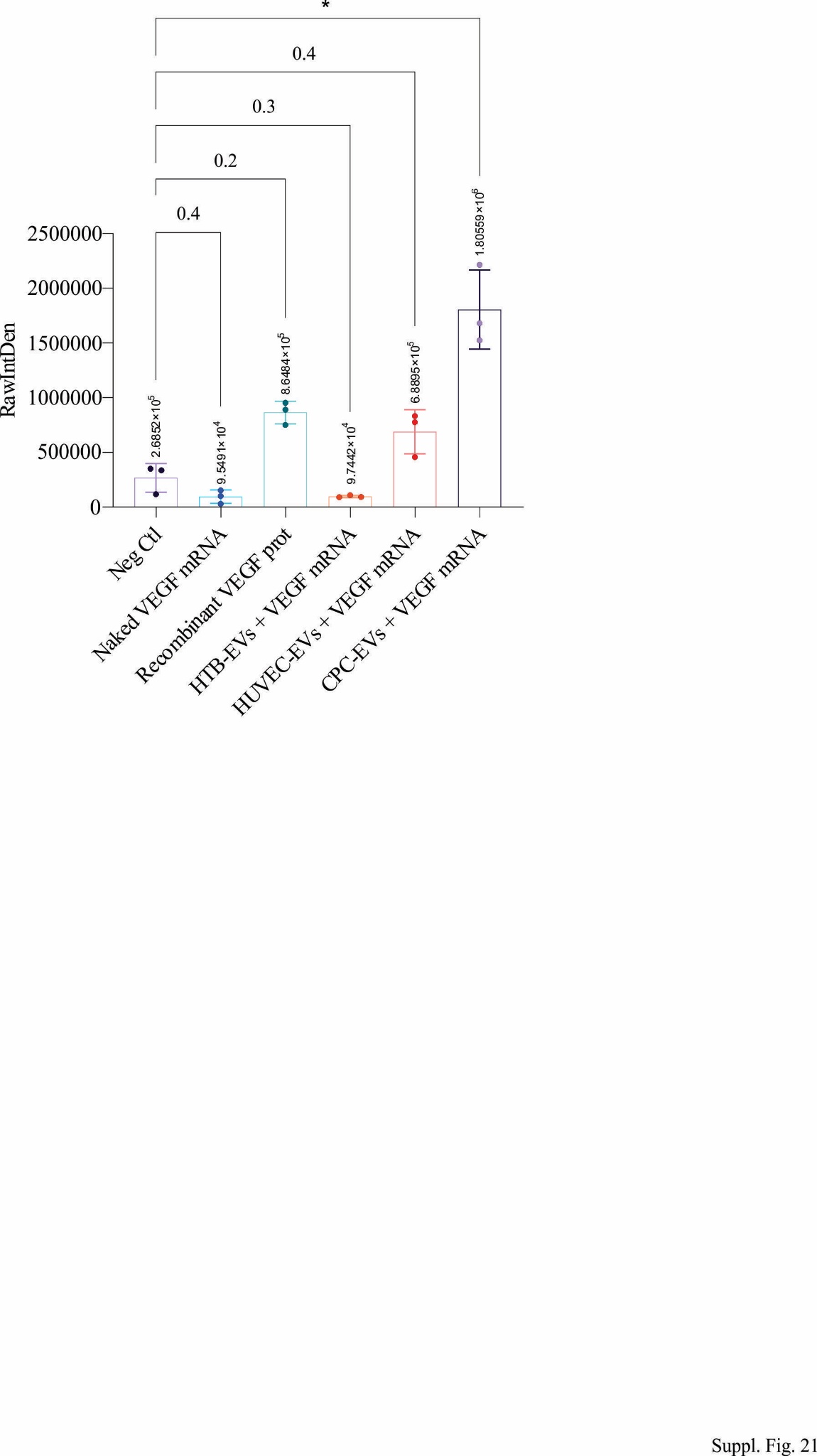


**Supplementary Figure 21. Quantification of aortic ring sprouting following *VEGF-A* mRNA delivery via EVs.** Quantitative analysis of aortic ring microscopy images was performed using ImageJ, a Java-based image processing program developed by the National Institutes of Health (NIH) and the Laboratory for Optical and Computational Instrumentation (LOCI, University of Wisconsin). Statistical comparisons among groups were performed using the Kruskal-Wallis test to compare treated and untreated samples. Statistical significance is indicated as *p < 0.05, whereas exact p-values are shown for non-significant comparisons. Data are presented as mean ± SD of n = 3. Neg Ctl: negative control (PBS). RawIntDen; Raw integrated density
